# Cytotoxic lymphocytes counteract viral type I interferon immune evasion

**DOI:** 10.1371/journal.ppat.1013955

**Published:** 2026-02-09

**Authors:** Michael Y. Schakelaar, Liling Shan, Shuang Li, Rianne G. Bouma, Josefien W. Hommes, Jorine G. F. Sanders, Jan Meeldijk, Laura L. Winkler, Toine ten Broeke, Robert F. Kalejta, Niels Bovenschen

**Affiliations:** 1 Department of Pathology, University Medical Center Utrecht, Utrecht, the Netherlands; 2 Center for Translational Immunology, University Medical Center Utrecht, Utrecht, the Netherlands; 3 Institute for Molecular Virology and McArdle Laboratory for Cancer Research, University of Wisconsin-Madison, Madison, Wisconsin, United States of America; Leibniz Institute of Virology (LIV), GERMANY

## Abstract

Viruses are recognized by host cell innate immunity through viral RNA/DNA sensing by cyclic GMP-AMP synthase (cGAS) stimulator of interferon genes (STING). However, many viruses evade cGAS-STING signaling and antiviral IFN-β response. Here, we show that natural killer (NK) cells counteract immune evasion of type I interferon response upon human cytomegalovirus (HCMV) infection. NK cells enhance IFN-β response in virus-infected cells more efficiently than perforin-knockout and GrM-knockout NK cells. Mechanistically, GrM cleaves viral pp71 into two fragments, the first, like full-length pp71, still inhibits cGAS-STING-IFN-β response but is rapidly degraded by the proteasome, and the second fragment that rather augments IFN-β and outperforms full-length pp71 inhibition of STING. NK cells cannot enhance IFN-β response in cells infected with HCMV that harbors a pp71 with a mutated GrM cleavage site. We conclude that NK cells use GrM to counteract cytomegaloviral innate immune evasion through pp71-mediated inhibition of cGAS-STING-IFN-β innate immune response.

## Author summary

Human cytomegalovirus (HCMV) is a common virus that can cause severe disease in newborns and people with weakened immune systems. To survive in the body, HCMV blocks an important early-warning system in infected cells called the cGAS-STING pathway, which normally triggers production of interferon-β to stop viral spread. We found that natural killer (NK) cells can overcome this viral defense. NK cells release an enzyme called granzyme M that cuts a viral protein, pp71, which is responsible for shutting down the interferon response. Once pp71 is cut, one piece is quickly destroyed and the other actually helps restore the antiviral signal. When the virus is engineered so pp71 cannot be cut, NK cells lose this ability to enhance interferon-β production. These results show that NK cells can “flip” a viral immune-evasion strategy and strengthen the body’s antiviral defenses, suggesting new ways to improve immunity against HCMV.

## Significance

To control viral infection, host cGAS-STING-IFN-β pathway plays a pivotal role in activating innate immunity and cytotoxic lymphocytes. Many viruses, are equipped with virulence molecules that block this pathway. Human cytomegalovirus (HCMV) is a life-long persisting β-herpes virus, present in >80% of the global population, that can cause serious disease and death. Here, we show that viral protein pp71 inhibits host type I interferon response after HCMV infection, and NK cells secrete serine protease granzyme M that enters infected cells through perforin pores to cleave and inactivate pp71, thereby restoring host cGAS-STING-IFN-β innate immune response. Our findings uncover a previously unrecognized non-cytotoxic antiviral function of NK cells, in which they overcome viral suppression of type I interferon responses—highlighting a conceptual shift in our understanding of how innate immunity restrains infection.

## Introduction

Cytotoxic lymphocytes (*e.g.,* CD8^+^ T cells and natural killer (NK) cells) are pivotal effector cells that control viral infections [1–4], mainly via the release of cytotoxic granules [1,5,6]. These granules contain perforin and a family of five structurally homologous serine proteases called granzymes (GrA, GrB, GrH, GrK, and GrM) [7–9]. While perforin facilitates the entry of granzymes into infected cells, granzymes can interfere with virus replication or kill the host cell [9–11].

The innate immune response and potent interferon (IFN-)β secretion are initiated by the cyclic GMP-AMP synthase (cGAS) stimulator of interferon genes (STING) pathway that detects pathogenic RNA/DNA upon viral infection of a host cell [12]. However, to counteract this innate immune response, several viruses – amongst those herpesviridae, poxiviridae, pappilomaviridae, adenoviridae, and coronaviridae – are equipped with virulence molecules that block cGAS-STING-IFN-β signaling [13,14]. Human cytomegalovirus (HCMV) belongs to the β-herpesvirus family and has a mean worldwide seroprevalence of about 80% that is even higher in individuals more likely to transmit the virus to vulnerable recipients (*e.g.,* women of reproductive age to neonates and blood and organ donors to recipients) [15]. HCMV infection is characterized by an asymptomatic lytic and a subsequent life-long latent infection in immunocompetent individuals [16,17]. However, HCMV reactivation from latency in immunocompromised patients, *e.g.,* in organ and stem cell transplant recipients and untreated AIDS patients, can cause devastating CMV disease [16,18]. In addition, HCMV infection is also a leading viral cause of congenital defects and may promote cancer [19–21].

HCMV Tegument phosphoprotein 71 (pp71) plays a pivotal role during infection in that it activates the major immediate early (IE) promoter to initiate the expression of IE genes and subsequent viral early and late proteins [22–26]. GrM is known to cleave pp71 and thereby inhibit HCMV IE expression and viral replication [27]. Pre- and post-infection treatment of fibroblasts with IFN-β was found to reduce HCMV production by over 99%, indicating that IFN-β alone is a potent inhibitor of viral replication [28]. HCMV is sensed by the cGAS-STING axis [29], but viral pp71 is an antagonist of the cGAS-STING pathway allowing HCMV to evade innate IFN-β antiviral immune response [30].

In the present study, we addressed the question whether NK cells can enhance host cell IFN-β innate immune response upon HCMV infection. Indeed, NK cells enhanced IFN-β response in HCMV-infected cells and this effect was not seen when infecting cells with mutant HCMV-pp71^L439A^, in which pp71 is resistant to GrM-mediated proteolysis. Consistent with this, perforin-knockout and GrM-knockout NK cells less efficiently enhanced IFN-β response in virus-infected cells as compared to wild-type NK cells. Our findings reveal a novel noncytotoxic function of NK cells to counteract viral inhibition of the cGAS-STING-IFN-β innate immune response.

## Results

### NK cells enhance innate IFN-β response in cytomegalovirus-infected cells

**Fig 1:**
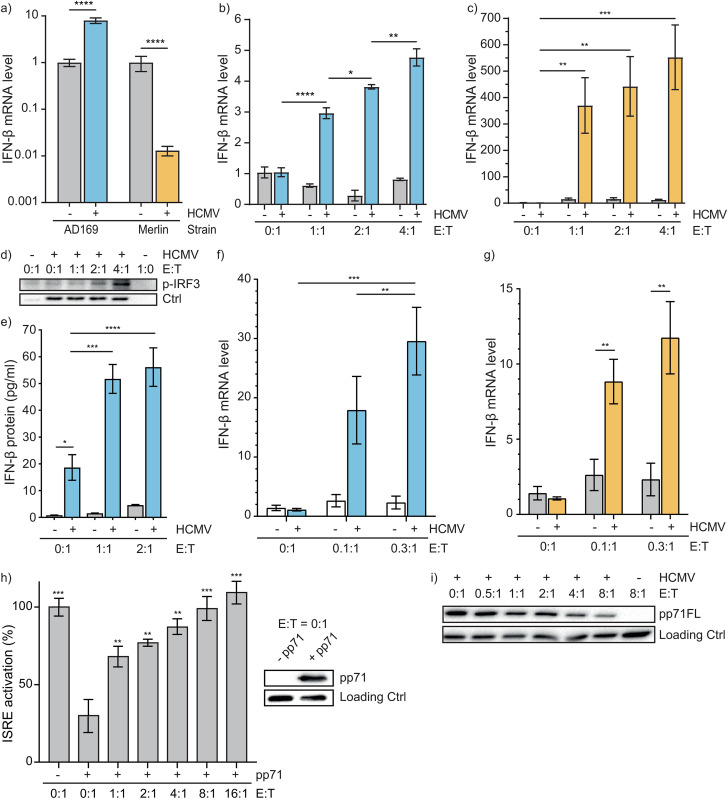
NK cells enhance innate IFN-β response in cytomegalovirus-infected cells. **(a)** HFFs were infected with GFP-HCMV-WT (AD169 [blue] or Merlin [orange], MOI = 0.2) for 2h and then maintained in medium for 18h. IFN-β mRNA levels were measured by qPCR. The value in the absence of virus was set to 1. Data is displayed as mean fold change ± SEM, n ≥ 3. **(b-c)** HFFs were infected with GFP-HCMV-WT (AD169 or Merlin, MOI = 0.2) for 2h and treated with NK cells (YT-Indy) at indicated E:T ratios for 18h. IFN-β mRNA levels were measured by qPCR. The value in the absence of NK cells was set to 1 to study the effect of NK cell treatment between the two virus strains. Data is displayed as mean fold change ± SEM, n ≥ 3. **(d)** HFFs were infected with GFP-HCMV-WT and co-cultured with NK cells at indicated E:T ratios for 18h. Fibroblasts were collected for immunoblotting for p-IRF3 and infection control (GFP). A representative blot is shown, n = 3. **(e)** HFFs were infected with GFP-HCMV-WT (AD169, MOI = 0.2) for 2h and co-cultured with NK cells (YT-Indy) at indicated E:T ratio for 18h. Supernatant was collected and tested for IFN-β protein by ELISA. Data is displayed as mean IFN-β concentration ± SD, n ≥ 3. **(f-g)** HFFs were infected with GFP-HCMV-WT (AD169 or Merlin, MOI = 0.2) for 2h and treated with primary NK cells at indicated E:T ratios for 18h. IFN-β mRNA levels were measured by qPCR. The value in the absence of primary NK cells was set to 1. Data is displayed as mean fold change ± SEM, n ≥ 3. **(h)** HeLa cells were transfected with cGAS and STING plasmids, together with pGL4.24 10 × ISRE reporter (100 ng), internal control hRL-EF1a (10 ng), and H2B-GFP (20 ng), in the presence or absence of pp71FL (410 ng), pp71FL expression is shown in the Western blot with loading control (GAPDH). 6h post-transfection, cells were treated with different amounts of NK cells (YT-Indy) for 18h. A dual luciferase reporter assay was used to measure ISRE activation. Relative luciferase activity, *i.e.,* firefly/renilla, in the absence of pp71 and NK cells was set to 100% and data were corrected for effects of NK cells in the absence of pp71. Data represent the mean ± SD, n = 3. **(i)** HFFs were infected with GFP-HCMV-WT (AD169, MOI = 0.2) for 24h and co-cultured with NK cells (YT-Indy) at indicated E:T ratios for 18h. Fibroblasts were lysed in reducing Laemmli buffer and subjected to immunoblotting for pp71FL and loading control (GAPDH). A representative blot is shown, n = 3. One-way ANOVA **(a-e)** was used for statistical analysis, * = p < 0.05, ** = p < 0.01, *** = p < 0.001, **** = p < 0.0001. E:T, effector:target; HCMV, human cytomegalovirus.

To investigate the effects of NK cells on the innate interferon response during HCMV infection, we first established IFN-β mRNA expression after infection of human foreskin fibroblasts (HFFs) with HCMV-AD169 (Fig 1a: blue) or HCMV-Merlin (Fig 1a: orange). We found an increase in IFN-β mRNA for HCMV-AD169-infected HFFs and a decrease for HFFs infected with HCMV-Merlin. Then, HCMV-AD169-infected (Fig 1b) and HCMV-Merlin-infected (Fig 1c) HFFs were treated with increasing amounts of NK cells and IFN-β mRNA was examined. Interestingly, host cell IFN-β mRNA increased markedly after NK cell treatment (Fig 1b-c, E(ffector):T(arget)=0:1 vs E:T = 1:1) and accumulated with higher E:T ratios (Fig 1b-c). Consistent with IFN-β mRNA, expression of p-IRF3 (Fig 1d) and IFN-β protein (Fig 1e) also increased after HCMV-AD169 infection which further accumulated with NK cell treatment. Similar effects on IFN-β mRNA were seen after treatment with primary NK cells isolated from healthy donor whole blood in both HCMV-AD169 (Fig 1f) and HCMV-Merlin-infected (Fig 1g) HFFs.

Since viral pp71 is an antagonist of the cGAS-STING-IFN-β pathway [30], we investigated the role of NK cells on viral pp71 in the cGAS-STING-IFN-β pathway. HeLa cells were transfected with cGAS, STING, and the IFN-stimulated response element (ISRE) reporter plasmid in the presence or absence of pp71, and these cells were subsequently treated with different amounts of NK cells (Fig 1h). Activation of ISRE was inhibited by pp71 in the absence of NK cells (Fig 1h, E:T = 0:1 – pp71FL vs E:T = 0:1 + pp71FL). Subsequent treatment with NK cells fully restored ISRE activation in a dose-dependent manner (Fig 1h). This effect coincided with a decrease in pp71FL (full length, Fig 1i) protein level in HCMV-infected HFF target cells following incubation with NK cells and dissipated with higher E:T ratios. Taken together, these data show that NK cells can increase IFN-β response in cells infected with different HCMV strains and pp71-expressing cells.

### NK cells restore cGAS-STING signaling and IFN-β response via GrM-mediated cleavage of pp71

**Fig 2:**
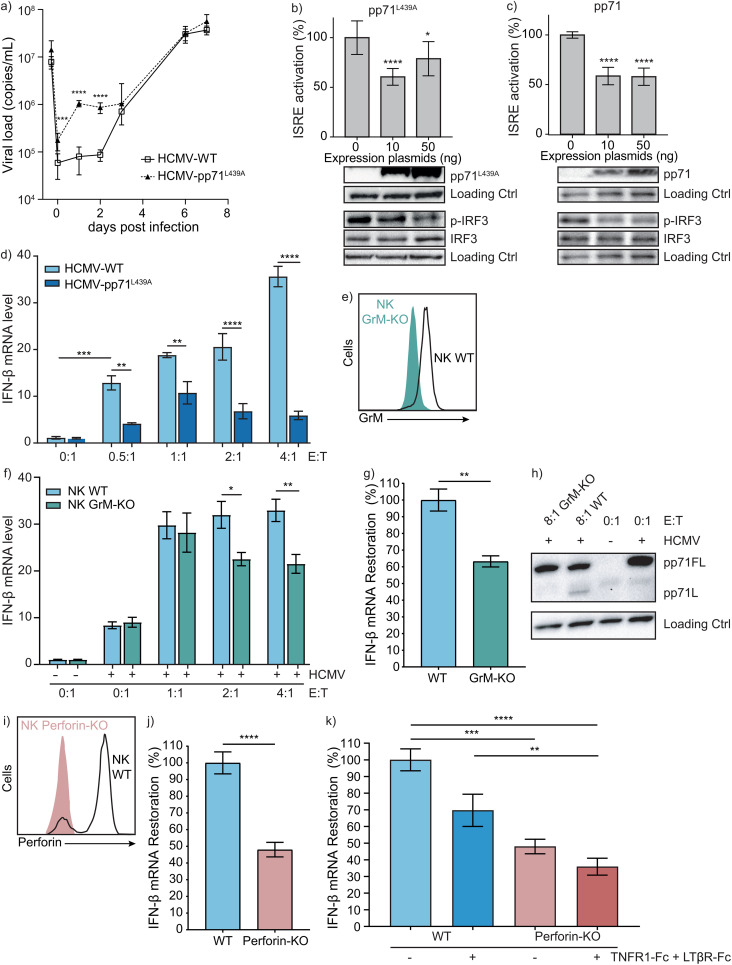
NK cells restore cGAS-STING signaling and IFN-β response via GrM-mediated cleavage of pp71 and signaling through TNFR1 and LTβR. **(a)** HFFs were infected with GFP-HCMV-WT or GFP-HCMV-pp71^L439A^ mutant (AD169, MOI = 0.2) for 2h. Viral load at indicated days post infection was measured by qPCR. Data represent the mean ± SD, n > 3. **(b-c)** HEK293T cells were transfected with pGL4.24 10 × ISRE (100 ng), hRL-EF1a (10 ng), c-Myc-cGAS (25 ng), Flag-STING (25 ng), H2B-GFP (30 ng), and increasing amounts of pp71^L439A^ **(b)** or pp71-WT **(c)**. 24h post-transfection, cells were lysed for dual luciferase reporter assay. Relative luciferase activity, *i.e.*, firefly/renilla, is depicted relative to the condition without pp71 (100%). Lysates were subjected to immunoblotting, using antibodies against HA, p-IRF3, IRF3, and loading control (Hsp90). Data represent the mean ± SD, n = 3. **(d)** HFFs were infected with GFP-HCMV-WT (light blue) or GFP-HCMV-pp71^L439A^ (dark blue) mutant (AD169, MOI = 0.2) and co-cultured with different amounts of NK cells (YT-Indy) for 18h. IFN-β mRNA was determined by qPCR. The value in the absence of NK cells was set to 1. Data represent the mean ± SEM, n = 3. **(e)** GrM-KO validation on flow cytometry and western blot with loading control (GAPDH). **(f)** HFFs were infected with GFP-HCMV-WT (AD169, MOI = 0.2) and co-cultured with different amounts of NK cells (WT (light blue) or GrM-KO (green) YT-Indy) for 18h. IFN-β mRNA was determined by qPCR. The value in the absence of virus and NK cells was set to 1. Data represent the mean ± SD, n = 3. **(g)** HFFs were infected with GFP-HCMV-WT (AD169, MOI = 0.2) and co-cultured with WT and GrM-KO NK cells (at E:T = 4:1) for 18h. IFN-β mRNA was determined by qPCR, WT NK cell IFN-β response was set at 100%. Data represent the mean ± SEM, n ≥ 3. **(h)** HFFs were infected with GFP-HCMV-WT (Merlin, MOI = 0.2) and co-cultured with WT and GrM-KO NK cells (at E:T = 8:1) for 18h. Cells were immunoblotted for pp71FL (and cleavage fragment) and loading control (GAPDH). Data shows representative blot, n = 3. **(i)** Perforin-KO validation on flow cytometry. **(j)** HFFs were infected with GFP-HCMV-WT (AD169, MOI = 0.2) and co-cultured with WT and GrM-KO NK cells (at E:T = 4:1) for 18h. IFN-β mRNA was determined by qPCR, WT NK cell IFN-β response was set at 100%. Data represent the mean ± SEM, n ≥ 3. **(k)** HFFs were infected with GFP-HCMV-WT (AD169, MOI = 0.2) and co-cultured with WT or Perforin-KO NK cells with or without 2h TNFR1-Fc and LTβR-Fc preincubation at E:T = 4:1 for 18h. IFN-β mRNA was determined by qPCR, WT NK cell IFN-β response without decoy proteins was set at 100%. Data represent the mean ± SEM, n ≥ 3. One-way ANOVA **(b-d,f,k)** or student t-test **(g,j)** was used for statistical analysis, * = p < 0.05, ** = p < 0.01, *** = p < 0.001, **** = p < 0.0001. Ctrl, control; E:T, effector:target; HCMV, human cytomegalovirus.

To elucidate the importance of GrM-mediated cleavage of pp71 in restoring IFN-β response, we have generated a mutant virus HCMV-pp71^L439A^, in which the putative GrM cleavage site Leu^439^ in pp71 has been mutated into alanine. Using this mutant virus in experiments with NK cells prerequisites that the mutation itself does not affect cGAS-STING-IFN-β signaling. Although the viral load of HCMV-pp71^L439A^ mutant increased significantly at the early time points (Fig 2a, day 0–2), the overall propagation kinetic was similar to that of HCMV-WT (Fig 2a, day 3–7). Additionally, pp71^L439A^ mutant protein still inhibited cGAS-STING-mediated ISRE activation with concomitant p-IRF3 inhibition (Fig 2b), *i.e.*, in a similar fashion as pp71-WT (Fig 2c). Next, NK cells were co-cultured with HCMV-pp71^L439A^ mutant-infected or HCMV-WT-infected HFFs that were subsequently analyzed for IFN-β mRNA. Whereas HCMV-WT-mediated inhibition of IFN-β production was relieved after NK cell addition, NK cells cannot fully restore IFN-β response in GrM-resistant HCMV-pp71^L439A^ mutant virus-infected HFFs (Fig 2d). To further substantiate the role for GrM, we knocked-out GrM (Fig 2e: green) in NK cells and treated HCMV-WT-infected HFFs with WT or GrM-knockout NK cells for IFN-β mRNA examination. GrM-knockout NK cells less efficiently restored IFN-β production in virus-infected cells at higher E:T ratios, as compared with WT-NK cells (Fig 2f). Moreover, GrM-KO NK cells restored ~60% of IFN-β signaling (Fig 2g). Additionally, immunoblotting for pp71 showed cleavage fragment pp71L upon co-culture with WT-NK cells but not GrM-KO NK cells (Fig 2h). To explore the function of other granzymes, we knocked-out perforin (Fig 2i, red) and co-cultured these NK cells with virus-infected HFFs. Perforin-KO NK cells restored ~45% of IFN-β mRNA (Fig 2j), indicating that other redundancy mechanism(s) still exist. Since tumor necrosis factor receptor 1 (TNFR1) and lymphotoxin β receptor (LTβR) on host cells are involved in the antiviral activity of NK cells [31,32], we investigated the effect of soluble decoy TNFR1-Fc and LTβR-Fc on IFN-β signaling. Indeed, IFN-β mRNA restoration by WT NK cells was decreased when TNFR1-Fc and LTβR-Fc were added (Fig 2k: blue). When Perforin-KO NK cells were used in combination with TNFR1-Fc and LTβR-Fc, IFN-β mRNA restoration was decreased to ~30% (Fig 2k: red). Collectively, these data indicate that NK cells restore IFN-β production in HCMV-infected cells in part via GrM-mediated cleavage of viral pp71.

### pp71FL and pp71S inhibit while pp71L activates IFN-β production

**Fig 3:**
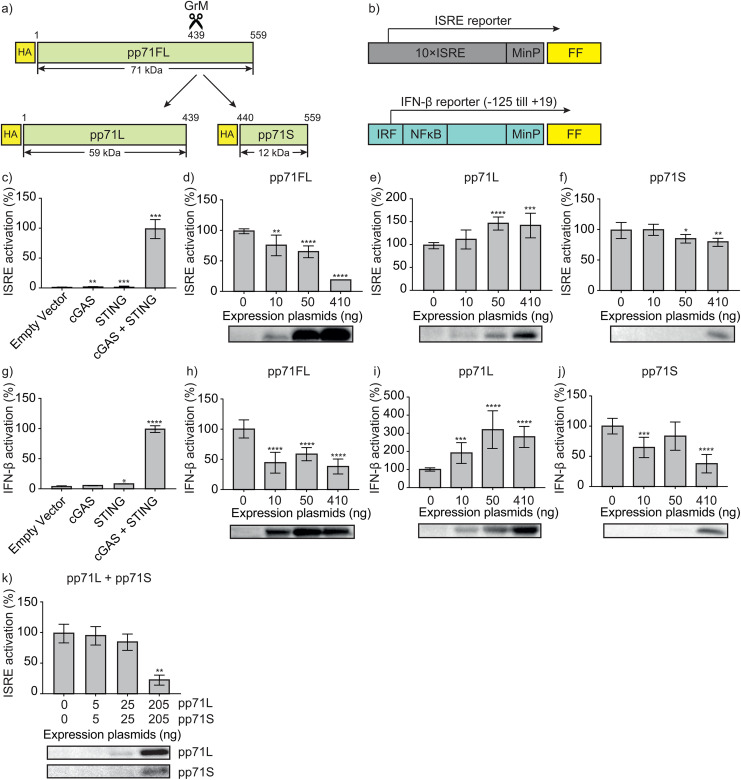
pp71FL and pp71S inhibit while pp71L activates IFN-β production. **(a)** Schematic overview of GrM-mediated cleavage of pp71FL into pp71L and pp71S. **(b)** Schematic overview of the ISRE and IFN-β promoter reporter constructs. **(c)** HeLa cells were transfected with ISRE reporter (100 ng), hRL-EF1a (10 ng), H2B-GFP (30 ng), and indicated plasmids. The relative luciferase activity in the condition of both cGAS and STING was defined as 100%. Data represent the mean ± SD, n = 3. **(d-f,k)** HeLa cells were transfected with ISRE reporter (100 ng), hRL-EF1a (10 ng), cGAS (25 ng), STING (25 ng), H2B-GFP (30 ng), and increasing amounts of pp71FL, pp71L and/or pp71S. Faint pp71S bands are a result of quick proteasomal degradation (see Fig 4f). Data represent the mean ± SD, n = 3. **(g)** HEK293T cells were transfected with IFN-β reporter (100 ng), hRL-EF1a (10 ng), H2B-GFP (30 ng) and indicated plasmids. The relative luciferase activity in the condition of both cGAS and STING was defined as 100%. Data represent the mean ± SD, n = 3. **(h-j)** HEK293T cells were transfected with IFN-β reporter (100 ng), hRL-EF1a (10 ng), cGAS (25 ng), STING (25 ng), H2B-GFP (30 ng), and increasing amounts of pp71FL, pp71L or pp71S. Faint pp71S bands are a result of quick proteasomal degradation (see Fig 4f). Data represent the mean ± SD, n = 3. Dual luciferase reporter assays were performed 24h after transfection. Lysates used in the luciferase reporter assay were subjected to immunoblotting using an anti-HA antibody. One-way ANOVA **(c-k)** was used for statistical analysis, * = p < 0.05, ** = p < 0.01, *** = p < 0.001, **** = p < 0.0001. FF, firefly; MinP, minimal promoter; pp71FL, pp71 full length; pp71L, pp71 long cleavage fragment; pp71S, pp71 short cleavage fragment.

To investigate the functional consequences of GrM-mediated cleavage of pp71 on IFN-β production, HA-tagged pp71FL, pp71L, and pp71S were constructed to mimic pp71 proteolysis by GrM (Fig 3a). A dual luciferase reporter assay was used to measure the effect of pp71 and its fragments on IFN-β reporter activation (Fig 3b). First, we used a 10 × ISRE reporter that was activated only when both cGAS and STING were expressed, as expected (Fig 3c). This ISRE activation was inhibited by pp71FL (Fig 3d) and pp71S (Fig 3f) in a dose-dependent manner. Surprisingly, pp71L did not inhibit but rather stimulated ISRE activation (Fig 3e). Second, we switched to a more physiologically relevant IFN-β reporter that contains the actual human promotor region for IFN-β transcription (Fig 3g). Again, pp71FL (Fig 3h) and pp71S (Fig 3j) inhibited IFN-β production, whereas pp71L (Fig 3i) stimulated IFN-β production. The combination of pp71L and pp71S fragments inhibited ISRE activation as well (Fig 3k). Apparently, simultaneous presence of pp71L and pp71S inhibited ISRE activation in a manner similar to pp71FL (Fig 3d). Thus, GrM cleaves pp71 into two fragments of which pp71S still inhibits IFN-β production and pp71L activates this innate immune response.

### pp71L reverses pp71FL function while pp71S is rapidly degraded by the proteasome

**Fig 4:**
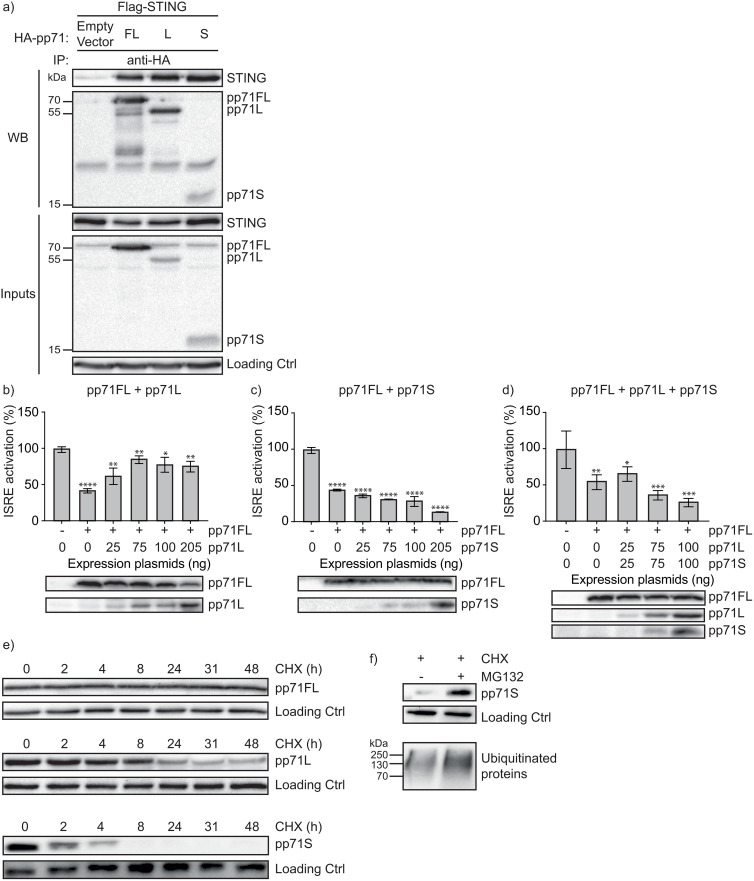
pp71L reverses pp71FL function while pp71S is rapidly degraded by the proteasome. **(a)** HEK293T cells were transfected with Flag-STING and HA-pp71FL/L/S for 24h. Cell lysates were incubated with anti-HA Sepharose beads and analyzed by immunoblotting with the indicated antibodies. **(b)** HEK293T cells were transfected with cGAS (400 ng), STING (400 ng), and pp71FL/L (400 ng) for 24h. Samples were subjected to immunoblotting using antibodies against pp71FL/L, flag-STING, p-IRF3, p-TBK-1, and loading control (HSP90). Densometry analysis was performed for p-IRF3 and p-TBK-1 in ImageJ and corrected for loading control. Data shows a representative immunoblot, n = 3 **(c-e)** HeLa cells were transfected with pGL4.24 10 × ISRE reporter (100 ng), hRL-EF1a (10 ng), cGAS (25 ng), STING (25 ng), H2B-GFP (30 ng), pp71FL (205 ng), and increasing amounts of pp71L and/or pp71S. Dual luciferase reporter assays were performed 24h after transfection. Lysates used in the luciferase reporter assay were subjected to immunoblotting using an anti-HA antibody. Data represent the mean ± SD, n = 3. **(f)** HEK293T cells were transfected with pp71FL, pp71L, pp71S (each 410 ng) and H2B-GFP (30 ng). 24h post-transfection, CHX (20 µM) was added for indicated time points. Samples were subjected to immunoblotting using antibodies against HA and loading/infection control (GFP). Data shows a representative immunoblot, n = 3 **(g)** HEK293T cells were transfected with pp71S and H2B-GFP. 24h after transfection, proteasome inhibitor MG132 (20 µM) and CHX (20 µM) were added to the cells for 4h. Cells were subjected to immunoblotting using anti-HA, loading/infection control (GFP), and ubiquitin antibodies. Data shows a representative immunoblot, n = 3. One-way ANOVA **(c-e)** was used for statistical analysis, * = p < 0.05, ** = p < 0.01, *** = p < 0.001, **** = p < 0.0001. Ctrl, control; pp71FL, pp71 full length; pp71L, pp71 long cleavage fragment; pp71S, pp71 short cleavage fragment.

It has been established that pp71 binds to STING, thereby blocking the recruitment of IRF-3 and TBK-1, leading to reduced IFN-β production [30]. We first tested whether GrM-generated pp71 cleavage fragments pp71L and pp71S can still bind to STING, using co-immunoprecipitation. Besides pp71FL, both pp71L and pp71S also interacted with STING (Fig 4a). Since pp71L activated IFN-β production (Fig 3e, i), we wondered whether this fragment could interfere with pp71FL thereby generating a negative feedback loop. Indeed, pp71L rescued pp71FL-mediated ISRE inhibition (Fig 4b), whereas pp71S alone (Fig 4c) or combination with pp71L (Fig 4d) augmented pp71FL function. Next, we performed a protein half-life test on pp71 and its fragments (Fig 4e) to study the stability of these proteins in the presence of the translation inhibitor cycloheximide (CHX). Protein levels of pp71FL remained stable during the time course of the experiment, while pp71L fragment displayed a half-life of approximately eight hours (Fig 4e). In sharp contrast, pp71S fragment was rapidly degraded, showed a half-life of less than two hours, and completely disappeared within eight hours after CHX addition (Fig 4e). To determine whether the degradation of pp71S is mediated by the proteasome, we added proteasome inhibitor MG132. In the presence of MG132, pp71S was successfully rescued alongside a panel of ubiquitinated proteins (Fig 4f). Our data indicate that GrM-mediated cleavage of pp71 results in a long pp71 cleavage fragment that short circuits pp71FL-mediated inhibition of IFN-β response and a short pp71 cleavage fragment that is rapidly degraded by the proteasome. In conclusion, NK cells use GrM to abolish viral pp71 function to enhance antiviral innate IFN-β immune response.

## Discussion

**Fig 5:**
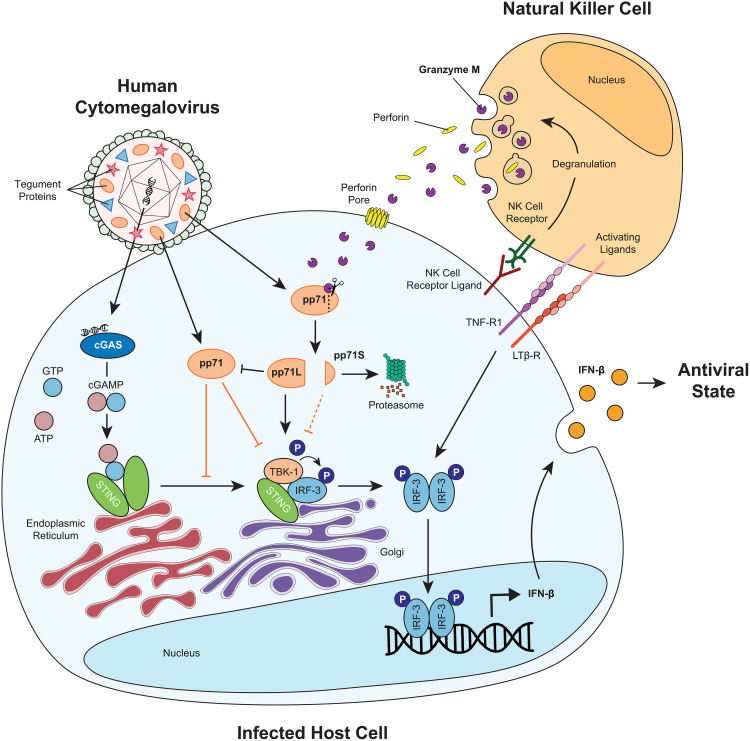
Schematic model. Graphical Abstract.

Viral pp71 is an antagonist of the cGAS-STING-IFN-β pathway, allowing HCMV to evade the innate immune response [30]. GrM, however, can cleave pp71 and inhibit HCMV IE expression and viral replication [27]. In the present study, we show for the first time that NK cells use perforin/GrM to non-cytotoxically enhance cGAS-STING pathway and IFN-β response in HCMV-infected cells via cleavage of viral tegument protein pp71 (Figs 1 and 2). NK cells do not adequately enhance IFN-β response in target cells infected with a mutant HCMV that lacks the GrM cleavage site in pp71 and GrM-/Perforin-KO NK cells less efficiently restore the IFN-β response as compared to wild-type NK cells (Fig 2). We further demonstrate that the long pp71 cleavage fragment does not inhibit but rather activates IFN-β production and outperforms pp71FL-mediated cGAS-STING signaling inhibition (Figs 3 and 4). Although pp71S still inhibits IFN-β production, it is rapidly degraded by the proteasome (Figs 3 and 4). Our data propose a novel, non-cytotoxic strategy developed by the immune system to counteract viral innate immune evasion inside host cells (Fig 5).

HCMV infection with AD169 or Merlin strain results in different IFN-β response (Fig 1). This can be explained by differential gene expression between HCMV-AD169 and HCMV-Merlin, *e.g.,* several NK evasion genes have been lost in AD169 whereas Merlin possesses the full-length genome [33].

NK cells do not enhance IFN-β response in HCMV-pp71^L439A^ mutant-infected cells (Fig 2). This raises the question why HCMV has not mutated pp71-Leu^439^ during evolution to evade GrM attack. This option becomes even more attractive when one considers our finding that the HCMV-pp71^L439A^ mutant virus grows slightly faster than HCMV-WT at earlier time points (Fig 2). However, the pp71-Leu^439^ residue is highly conserved (S1 Fig) among different HCMV strains (*e.g.,* AD196, Merlin, TB40e, FIX, TR, Towne, ten non-passaged patient strains, and a chimp HCMV strain [34]) in primates but not in mice, suggesting a primate-specific evolutionary advantage of Leu^439^ over an alternative amino acid. The enhanced early replication of the pp71 L439A mutant could reflect increased stability of this cleavage-resistant protein, which prolongs its activity in initiating MIE promoter activation; even modest early differences are amplified by the IE1 positive-feedback circuit, resulting in higher titres at 1–2 dpi. However, further research is required to investigate the importance of Leu^439^ in pp71, teaching us why HCMV maintains this residue.

Cytotoxic cells (*e.g.,* CD8^+^ T cells and NK cells) arrive within hours at HCMV-infected cells to participate in viral clearance by producing antiviral cytokines and by killing of virus-infected cells [35–37]. If cytotoxic cells were to kill HCMV-infected cells before recovering the IFN-β response, the relevance of restoring cGAS-STING-IFN-β signaling by cytotoxic cells would be questionable. However, HCMV has developed a variety of strategies to avoid host cell killing by expression of inhibitors of apoptosis, including UL122, UL123, UL36, UL37 [38]. In addition, Halle and co-workers have recently demonstrated that the *in vivo* killing capacity of cytotoxic T cells is limited and that multiple hits by cytotoxic cells are needed to kill a single CMV-infected cell [39]. Hence, cytotoxic cells may enhance the antiviral cGAS-STING-IFN-β response to induce an antiviral state and limit HCMV infection before accomplishing host cell killing.

Apparently, cytotoxic cells can interfere with viral type I interferon immune evasion. In line with this, it has been demonstrated that activated NK cells that express lymphotoxin (LT)αβ and secrete LTα and TNF can extracellularly activate the canonical (p65/p50 dependent) NF-κB pathway, resulting in enhanced host cell IFN-β mRNA expression and inhibited HCMV replication [31,32] (Fig 2). In the present study, we further reveal that NK cells can enhance IFN-β response in HCMV-infected cells via perforin-delivered GrM and GrM-mediated cleavage of viral pp71 inside infected cells (Figs 1 and 2). Furthermore, we show that other human granzymes (*i.e.,* GrA, GrB, GrH, and/or GrK) also contribute to IFN-β expression upon HCMV-infection (Fig 2: Perforin-KO) but it remains unclear if this is through targeting pp71. Additionally, TNFR1/LTβR signaling may regulate the status and activation of NK cells, *i.e.*, modulate the production of granzymes. Additionally, other molecular mechanisms, beside granzymes and TNFR1/LTβR signaling, could be employed to restore pp71-mediated IFN-β inhibition. Moreover, besides pp71, several other HCMV-encoded proteins have recently been identified to interfere with the cGAS-STING-IFN-β axis. Of these, pp65 and UL31 are involved in cGAS inactivation, IE2 induces STING degradation [40–42], and UL138 inhibits IFN-β response downstream of STING [43]. Whether these viral proteins could also be targeted by cytotoxic cell-derived GrM or other granzymes, thereby further contributing to enhance IFN-β response, remains to be investigated. Finally, also granzyme- and STING-independent mechanisms may contribute to cytotoxic cell-mediated IFN-β regulation in host cells, *e.g.,* via TNF-related cytokines that are known players in NF-κB-dependent induction of IFN-β [31, 14, 44].

It has been shown that pp71 residues 194–372 mediate binding to STING, thereby inhibiting IFN-β response [30]. Consistently, we found that GrM-induced pp71L (residues 1–439) fragment binds to STING (Fig 4). Production of IFN-β is not inhibited by pp71L, but pp71L predominates over pp71FL and activates cGAS-STING-IFN-β signaling (Figs 3–4). This suggests that pp71L may interfere with pp71FL binding to STING or may prevent STING trafficking, resulting in a negative feedback loop to further reduce IFN-β inhibition upon pp71 proteolysis by GrM. Hence, in a system with only pp71L, cGAS, and STING, we observe an increase in IFN-β reporters suggesting that – besides outperforming pp71FL – pp71L alone can activate cGAS-STING-IFN-β signaling. This opens the possibility that pp71L can be employed as therapeutic option as agonist for the cGAS-STING axis. In addition, targeting pp71 by GrM may provide a novel antiviral immunotherapeutic strategy. Increasing GrM expression in cytotoxic cells not only augments the inhibition of viral replication [27], but also may enhance the antiviral cGAS-STING-IFN-β response. These therapeutic strategies could be combined with emerging STING agonists, which have already been shown to reduce herpes simplex virus 2 replication in mice [45].

HCMV infection initiates the cGAS-STING-IFN-β pathway through recognition of viral dsDNA. pp71FL inhibits the cGAS-STING-IFN-β pathway through inhibition of STING trafficking and direct binding to STING. NK cells get activated by NK cell receptors and release granzymes and perforin (degranulation). Furthermore, NK cells activate IFN-β production through TNFR1 and LTβR signaling. Perforin forms a pore in the membrane of the virus-infected cell and allows Granzyme M to enter. After Granzyme M-mediated cleavage of pp71, pp71S is still able to inhibit IFN-β production, but is rapidly degraded by the proteasome. pp71L can no longer inhibit IFN-β production, further cancels out STING inhibition by pp71FL, and even activates IFN-β pathway.

## Materials and methods

### Cell culture and transfection

Cells were cultured in a 5% CO_2_ atmosphere at 37˚C. Human foreskin fibroblasts (HFFs), HeLa cells, and human embryonic kidney (HEK293T) cells were maintained in Dulbecco’s modified Eagle medium (DMEM, Gibco) and NK cell line YT-Indy was cultured in RPMI 1640 medium (Gibco), supplemented with 10% fetal calf serum (FCS, BioWest), 100 μg/mL streptomycin and 100 units/mL penicillin (Invitrogen), as described in [46]. Transfection of all plasmids was done using polyethylenimine (PEI, Polysciences Inc.) in a 3:1 ratio with DNA.

### Primary NK cells

Peripheral blood mononuclear cells (PBMCs) were isolated from healthy human donors (UMC Utrecht, Mini Donor Dienst) by Ficoll-Paque gradient centrifugation. Subsequently, negative selection NK-isolation was performed using a magnetic cell separation system (MACS) with a human NK cell isolation kit (Miltenyi Biotec, 130-092-657). Populations of enriched primary NK cells typically contained 80 – 90% CD56^+^ cells, confirmed by flow cytometry. Primary NK cells were not cultured or stimulated and directly used in experimental co-cultures after isolation.

### Viruses, antibodies, and reagents

GFP-tagged HCMV-WT (AD169) virus was generated as previously described [47]. GFP-tagged HCMV-WT (Merlin) virus was a generous gift from Prof. Dr. R. Stanton (Cardiff University, UK). To create HCMV-pp71L439A mutant virus, bacterial artificial chromosome (BAC)-derived AD169-GFP was mutagenized using a two-step red recombination system [48]. HCMV pp71 codon 439, CTG coding for leucine, was replaced with GCG coding for alanine. The oligonucleotide sequences used for mutagenesis were: pp71^L439A^ forward: 5’-GACGACGAAGATGACCTCTCCTCCACACCGACGCCGACCCCCGCGTCCGAAGCCATGTTTGCCGGCTAGGGATAACAGGGTAATCGATTT-3’; pp71^L439A^ reverse: 5’-GTCGCCGCTGGCTTCCTCGAAGCCGGCAAACATGGCTTCGGACGCGGGGGTCGGCGTCGGGCCAGTGTTACAACCAATTAACC-3’. Nucleotide substitution was confirmed by sequencing. Virus was reconstituted from BACs by transfection as previously described [49] and confirmed by sequencing. Primary antibodies used for the following proteins were obtained from commercial sources: GFP (mouse monoclonal, 7.1 and 13.1, Roche, 1:1000), p-IRF3 (rabbit monoclonal, 4D4G, Cell Signaling Technology (CST), 1:1000), p-TBK-1 (rabbit monoclonal, D52C2, CST, 1:1000) IRF3 (mouse monoclonal, SL-12, Santa Cruz Biotechnology, 1:1000), Hsp90 (mouse monoclonal, K3701, Enzo Life Sciences, 1:1000), GAPDH (mouse monoclonal, 6C5, Abcam, 1:5000), Flag (mouse monoclonal, M2, Sigma-Aldrich, 1:1000), ubiquitin (mouse monoclonal, Ubi-1, Merck), and GrM (mouse monoclonal, 4D11, NOVUS Biologicals, 1:250). The antibodies for HA tag (12CA5, 1:500) and pp71FL (IE233, 1:300) were mouse hybridoma supernatants. Secondary HRP-conjugated goat anti-mouse/rabbit antibodies were from Jackson ImmunoResearch Laboratories (1:5000). Proteasome inhibitor MG132 and CHX were from Sigma.

### Plasmids

Mammalian expression vector pCGN-HA-pp71 was a generous gift from Prof. Dr. T. Shenk (Princeton University, USA). Generation of HA-pp71^L439A^ mutant, HA-pp71^1-439^ and HA-pp71^440-559^ constructs have been described previously [27]. Flag-STING and c-Myc-cGAS were from IDT (Gblocks), and subcloned into pcDNA3.1-Puro vector using standard molecular biology techniques. pGL4.24 10 × ISRE was generated by insertion of a tandem repeat of 5 × ISRE in pGL4.24 (Promega) between Eco53kI and EcoRV sites. 5 × ISRE was produced using forward primer 5’-GAGCTCTAGTTTCACTTTCCC-3’, reverse primer 5’-ATCCTCGAGGGGAAAGTG-3’ and 5’-GAGCTCTAGTTTCACTTTCCCTAGTTTCACTTTCCCTAGTTTCACTTTCCCTAGTTTCACTTTCCCTAGTTTCACTTTCCCCTCGAGGAT-3’ as template. IFN-β reporter pGL4.11-IFN-β [-125 till +19] [50] was obtained using forward primer 5’-ATCAAGGACCAACTGTATCTTTTAGTG-3’, reverse primer 5’-CTCGAGTCAGTTTCGGAGGTAACC-3’, and human chromosomal DNA as template, subcloned into pGL4.11 (Promega) by digestion with HindIII and EcoRV-HF. All constructs were verified by sequencing.

### Generation of GrM-/Perforin-KO NK cell lines by CRISPR/Cas9

NK cell line YT-Indy was electroporated with Neon Transfection System (Thermo Fisher Scientific). Basic steps were performed as IDT manufacturer’s protocol described for delivery of ribonucleoprotein complexes into Jurkat T cells. Briefly, equal amounts of crRNA and tracrRNA were mixed before adding Cas9 to form the RNP complex. After 20 minutes incubation, cells were electroporated and grown in a 96-wells flat bottom plate. After 72 hours, the knockout efficiency was verified by flow cytometry, PCR, and Sanger sequencing. As described previously [46], Limiting dilution was used to generate single-cell clones of GrM-/Perforin-KO NK cells that were confirmed by flow cytometry. All steps were performed at room temperature. Two crRNAs were combined for higher targeting efficiency. The GrM gRNA#1 target sequence was 5’-TTTGGGACCCAGATCATCGG-3’. gRNA#2 target sequence was 5’-TGTCCTGGTGCACCCAAAGT-3’.

### HCMV infection model

Target HFF cells were seeded in 24-wells plates and infected with GFP-HCMV-WT (AD169 or Merlin) or GFP-HCMV-pp71^L439A^ mutant at a multiplicity of infection (MOI) of 0.2 for 2 hours at 37˚C. Cells were washed twice with serum-free DMEM and cultured in 500 µl complete DMEM medium. To measure viral load by qPCR, supernatant was collected at the indicated time points and HCMV was inactivated for 30 minutes at 60˚C. For co-culture experiments, NK cells were added immediately after washing twice with serum-free DMEM at different E:T ratios and co-cultured for 18 hours in the presence of ZVAD-fmk (100 µM). For decoy protein experiment, TNFR1-Fc (1 ug/ml) and LTβR-Fc (2 ug/ml) was added to NK cells 2h before starting the co-culture. For qPCR analysis of IFN-β mRNA level, cells were washed at least five times with ice-cold PBS and directly lysed in TRIzol reagent (Thermo Fisher) and stored at -80°C until qPCR analysis. For secreted IFN-β protein analysis, supernatant was collected, spun down at 500×g to clear cell debris, and stored at -20°C until further analysis. For endogenous protein level analysis, cells were washed at least five times with PBS and directly lysed in reducing Laemmli sample buffer (0.125 M Tris HCl (pH=6.8), 4% SDS, 20% glycerol, 0.004% bromophenol blue, 200 mM DTT), boiled for 5 minutes, and stored at -20°C until immunoblotting. We cannot differentiate between IFN-β production by infected cells or bystander cells because of the MOI and time of infection. After washing, removal of NK cells was microscopically confirmed. The IFN-β mRNA from left-over NK cells are not likely to have contributed to the observed effects because IFN-β mRNA in NK cells is inhibited in the presence of HCMV (S2 Fig).

### NK-HeLa cell co-culture

Two transfection mixtures were prepared, both consisting of cMyc-cGAS, Flag-STING, pGL4.24 10 × ISRE, hRL-EF1a, and GFP, either with HA-pp71FL or with empty vector. HeLa cells were transfected using PEI. After 6 hours of transfection, NK cells were added at indicated E:T ratios for 18 hours co-culture at 37˚C in the presence of ZVAD-fmk (100 µM). Cells were lysed for a dual luciferase reporter assay using 100 μL passive lysis buffer (Promega).

### qPCR

Total RNA was isolated using TRIzol reagent (Thermo Fisher) according to manufacturer’s protocol. cDNA was synthesized from total RNA (1.5 µg) using SuperScript reverse transcriptase (Invitrogen) and amplified using TaqMan universal PCR master mix (Applied Biosystems). TaqMan gene expression assays for IFN-β1 (Hs01077958_s1) and glyceraldehyde-3-phosphate dehydrogenase (GAPDH, Hs03929097_g1) were from Applied Biosystems. The real-time qPCR reactions were ran on a ViiA 7 standard real-time PCR system (Applied Biosystems). IFN-β1 expression value was normalized against the GAPDH endogenous control. The relative gene expression ratios were obtained using the 2^-ΔΔCT^ method. Viral replication measurement for GFP-HCMV-WT/pp71^L439A^ mutant was performed as described previously [51].

### ELISA IFN-β

ELISA assay (pbl assay science, 41410–1) was performed according to the manufacturer’s instructions. A standard curve was calculated (R^2^ = 0.9953) that was used to determine equivalent sample IFN-β concentrations.

### Dual luciferase reporter assays

HeLa or HEK293T cells were seeded in 24-well plates and transfected with H2B-GFP (15 ng), 25 ng c-Myc-cGAS, and Flag-STING, indicated amounts of pCGN-HA-pp71 (pp71FL), pCGN-HA-pp71^L439A^ mutant (pp71L439A), pCGN-HA-pp71^1-439^ (pp71L), pCGN-HA-pp71^440-559^ (pp71S), 100 ng of pGL4.11-IFN-β [-125 till +19] or pGL4.24 10 × ISRE, and 10 ng hRL-EF1a. For combination assays, HeLa cells were transfected with ISRE reporter (100 ng), hRL-EF1a (10 ng), cGAS (25 ng), STING (25 ng), H2B-GFP (30 ng), and increasing amounts of pp71L and pp71S as indicated. For competition assays, HeLa cells were transfected with pGL4.24 10 × ISRE reporter (100 ng), hRL-EF1a (10 ng), cGAS (25 ng), STING (25 ng), H2B-GFP (30 ng), pp71FL (205 ng), and increasing amounts of pp71L or/and pp71S as indicated. 24 hours post-transfection, cells were lysed using 100 μL passive lysis buffer (Promega). Firefly and renilla luciferase activities were determined with Dual-Luciferase Reporter Assay System (Promega) in a Veritas Microplate Luminometer (Turner Biosystems) according to the manufacturer’s protocol (Promega). Both luciferase activities were corrected for background signal and subsequently firefly activity was normalized by renilla internal control. To determine protein expression levels, cell lysates were subjected to immunoblotting with an anti-HA antibody.

### Protein half-life assay

HEK293T cells were seeded in 24-well plates and transfected with 410 ng pCGN-HA-pp71 (pp71FL), pCGN-HA-pp71^1-439^ (pp71L), pCGN-HA-pp71^440-559^ (pp71S) together with 30 ng H2B-GFP for 24 hours. CHX (20 µM) was added subsequently and incubated for indicated time points. For the pp71S rescue experiment, MG132 (20 µM) was added together with CHX (20 μM) for 4 hour incubation. Afterwards cells were directly lysed in reducing Laemmli sample buffer and subjected to immunoblotting.

### Immunoprecipitation

HEK293T cells were seeded in 10 cm dishes and transfected with 200 ng GFP, 5000 ng Flag-STING and 5000 ng empty vector or HA-tagged pCGN-71 (pp71FL), pCGN-71^1-439^ (pp71L), pCGN-71^440-559^ (pp71S). After 24 hours, cells were washed with PBS and lysed in NP40 lysis buffer (20mM Tris-HCl, 150mM NaCl, 1 mM EDTA, 1% Nonidet P40, protease inhibitor (11873580001, Roche), phosphatase inhibitor (04906837001, Roche)). Supernatants were collected after centrifuge at 10,000 × g for 10 minutes at 4°C. Then, 500 µl cleared cell lysates were incubated with 25 µl anti-HA agarose beads (11815016001, Roche) overnight at 4°C end over end. Beads were separated from the supernatant by centrifugation and washed four times with NP40 lysis buffer. Reducing Laemmli sample buffer was added to beads and boiled for 5 min at 95°C before immunoblotting.

### Statistical analysis

Statistical analysis was performed in GraphPad Prism using Student t-test and one-way ANOVA after checking data for normality using Shapiro-Wilk test. *P-*values of less than 0.05 were considered to be significant. Statistical details of experiments are described in the Fig legends.

## Supporting information

S1 Fig MUSCLE analysis. Homology analysis of pp71.
(PDF)

S2 Fig HCMV Infection of HFF and NK cells. HFF or NK cells were incubated with or without HCMV-AD169 (MOI = 0.2) for 2 hours and cultured overnight. Then, mRNA for IFN-β was measured by qPCR with HFF only (without virus) set at 1.
(PDF)

S3 Fig Raw data. All data used to make the Figs, also including full immunoblots.
(PDF)
