# Supplementary material for: Cytotoxic lymphocytes counteract viral type I interferon immune evasion
Source: PLoS Pathog. 2026 Feb 9;22(2):e1013955. doi: 10.1371/journal.ppat.1013955 (PMC12912695; doi:10.1371/journal.ppat.1013955)
Supplement: S1 Fig — Homology analysis of pp71. (PDF) [file ppat.1013955.s001.pdf]

S1 Fig Aligned using MUSCLE

```
Consensus              MSQASSSPGEGPSSEAAAISEAEAASGSFGRLHCQVLRLITNVEGGSLEAGRLRLLDLRTNIEVSRPSVLCCFQENKSPH
                                10        20        30        40        50        60        70        80
BE212010_UL82          MSQASSSPGEGPSSEAAAISEAEAASGSFGRLHCQVLRLITNVEGGSLEAGRLRLLDLRTNIEVSRPSVLCCFQENKSPH  80
HANChild4_UL82         MSQASSSPGEGPSSEAAAISEAEAASGSFGRLHCQVLRLITNVEGGSLEAGRLRLLDLRTNIEVSRPSVLCCFQENKSPH  80
HANRTR2_UL82           MSQASSSPGEGPSSEAAAISEAEAASGSFGRLHCQVLRLITNVEGGSLEAGRLRLLDLRTNIEVSRPSVLCCFQENKSPH  80
HANRTR4_UL82           MSQASSSPGEGPSSEAAAISEAEAASGSFGRLHCQVLRLITNVEGGSLEAGRLRLLDLRTNIEVSRPSVLCCFQENKSPH  80
HANRTR5_UL82           MSQASSSPGEGPSSEAAAISEAEAASGSFGRLHCQVLRLITNVEGGSLEAGRLRLLDLRTNIEVSRPSVLCCFQENKSPH  80
HANSCTR1B_UL82         MSQASSSPGEGPSSEAAAISEAEAASGSFGRLHCQVLRLITNVEGGSLEAGRLRLLDLRTNIEVSRPSVLCCFQENKSPH  80
HANSCTR4_UL82          MSQASSSPGEGPSSEAAAISEAEAASGSFGRLHCQVLRLITNVEGGSLEAGRLRLLDLRTNIEVSRPSVLCCFQENKSPH  80
UKLon1Blood2013_UL82   MSQASSSPGEGPSSEAAAISEAEAASGSFGRLHCQVLRLITNVEGGSLEAGRLRLLDLRTNIEVSRPSVLCCFQENKSPH  80
UKLon6Urine2011_UL82   MSQASSSPGEGPSSEAAAISEAEAASGSFGRLHCQVLRLITNVEGGSLEAGRLRLLDLRTNIEVSRPSVLCCFQENKSPH  80
PAV1_UL82              MSQASSSPGEGPSSEAAAISEAEAASGSFGRLHCQVLRLITNVEGGSLEAGRLRLLDLRTNIEVSRPSVLCCFQENKSPH  80
Lab_AD169_UL82         MSQASSSPGEGPSSEAAAISEAEAASGSFGRLHCQVLRLITNVEGGSLEAGRLRLLDLRTNIEVSRPSVLCCFQENKSPH  80
Lab_FIX-UL82           MSQASSSPGEGPSSEAAAISEAEAASGSFGRLHCQVLRLITNVEGGSLEAGRLRLLDLRTNIEVSRPSVLCCFQENKSPH  80
Lab_Merlin-UL82        MSQASSSPGEGPSSEAAAISEAEAASGSFGRLHCQVLRLITNVEGGSLEAGRLRLLDLRTNIEVSRPSVLCCFQENKSPH  80
Lab_TB40e-UL82         MSQASSSPGEGPSSEAAAISEAEAASGSFGRLHCQVLRLITNVEGGSLEAGRLRLLDLRTNIEVSRPSVLCCFQENKSPH  80
Lab_Towne_UL82         MSQASSSPGEGPSSEAAAISEAEAASGSFGRLHCQVLRLITNVEGGSLEAGRLRLLDLRTNIEVSRPSVLCCFQENKSPH  80
Lab_TR_UL82            MSQASSSPGEGPSSEAAAISEAEAASGSFGRLHCQVLRLITNVEGGSLEAGRLRLLDLRTNIEVSRPSVLCCFQENKSPH  80
Lab_Herberling-UL82    MSR-SPSPGEGPSAAGGPGGAPGDNGSTFGRMHCQVLRLVTNHD-SSLEPDRLKILDLRTSVEVSRTSVLCLFQENKSQH  78

Consensus              DTVDLTDLNIKGRCVVGEQDRLLVDLNNFGPRRLTPGSENNTVSVLAFALPLDRVPVSGLHLFQSQRRGGEENRPRXEAR
                                90       100       110       120       130       140       150       160
BE212010_UL82          DTVDLTDLNIKGRCVVGEQDRLLVDLNNFGPRRLTPGSENNTVSVLAFALPLDRVPVSGLHLFQSQRRGGEENRPRMEAR  160
HANChild4_UL82         DTVDLTDLNIKGRCVVGEQDRLLVDLNNFGPRRLTPGSENNTVSVLAFALPLDRVPVSGLHLFQSQRRGGEENRPRVEAR  160
HANRTR2_UL82           DTVDLTDLNIKGRCVVGEQDRLLVDLNNFGPRRLTPGSENNTVSVLAFALPLDRVPVSGLHLFQSQRRGGEENRPRMEAR  160
HANRTR4_UL82           DTVDLTDLNIKGRCVVGEQDRLLVDLNNFGPRRLTPGSENNTVSVLAFALPLDRVPVSGLHLFQSQRRGGEENRPRMEAR  160
HANRTR5_UL82           DTVDLTDLNIKGRCVVGEQDRLLVDLNNFGPRRLTPGSENNTVSVLAFALPLDRVPVSGLHLFQSQRRGGEENRPRMEAR  160
HANSCTR1B_UL82         DTVDLTDLNIKGRCVVGEQDRLLVDLNNFGPRRLTPGSENNTVSVLAFALPLDRVPVSGLHLFQSQRRGGEENRPRMEAR  160
HANSCTR4_UL82          DTVDLTDLNIKGRCVVGEQDRLLVDLNNFGPRRLTPGSENNTVSVLAFALPLDRVPVSGLHLFQSQRRGGEENRPRMEAR  160
UKLon1Blood2013_UL82   DTVDLTDLNIKGRCVVGEQDRLLVDLNNFGPRRLTPGSENNTVSVLAFALPLDRVPVSGLHLFQSQRRGGEENRPRVEAR  160
UKLon6Urine2011_UL82   DTVDLTDLNIKGRCVVGEQDRLLVDLNNFGPRRLTPGSENNTVSVLAFALPLDRVPVSGLHLFQSQRRGGEENRPRVEAR  160
PAV1_UL82              DTVDLTDLNIKGRCVVGEQDRLLVDLNNFGPRRLTPGSENNTVSVLAFALPLDRVPVSGLHLFQSQRRGGEENRPRMEAR  160
Lab_AD169_UL82         DTVDLTDLNIKGRCVVGEQDRLLVDLNNFGPRRLTPGSENNTVSVLAFALPLDRVPVSGLHLFQSQRRGGEENRPRMEAR  160
Lab_FIX-UL82           DTVDLTDLNIKGRCVVGEQDRLLVDLNNFGPRRLTPGSENNTVSVLAFALPLDRVPVSGLHLFQSQRRGGEENRPRMEAR  160
Lab_Merlin-UL82        DTVDLTDLNIKGRCVVGEQDRLLVDLNNFGPRRLTPGSENNTVSVLAFALPLDRVPVSGLHLFQSQRRGGEENRPRMEAR  160
Lab_TB40e-UL82         DTVDLTDLNIKGRCVVGEQDRLLVDLNNFGPRRLTPGSENNTVSVLAFALPLDRVPVSGLHLFQSQRRGGEENRPRVEAR  160
Lab_Towne_UL82         DTVDLTDLNIKGRCVVGEQDRLLVDLNNFGPRRLTPGSENNTVSVLAFALPLDRVPVSGLHLFQSQRRGGEENRPRVEAR  160
Lab_TR_UL82            DTVDLTDLNIKGRCVVGERDRLLVDLNNFGPRRLTPGSENNTVSVLAFALPLDRVPVSGLHLFQSQRRGGEENRPRMEAR  160
Lab_Herberling-UL82    DTVDLTDLNVKGHCAVGERDQLKADLINYSQRRMSPGS-STPISVLAFGLPLERVPVSGIHLFQAHPRGDEENRLRTEAR  157
```

**Consensus**	**AIIRRTAHXWAVRLTVTPNWRRRTDSSLEAGQIFVSQFAFRAGAIPLTLVDALEQLACSDPNTYIHKTETDERGQWIMLF**	
	170 180 190 200 210 220 230 240	
BE212010_UL82	AIIRRTAHHWAVRLTVTPNWRRRTDSSLEAGQIFVSQFAFRAGAIPLTLVDALEQLACSDPNTYIHKTETDERGQWIMLF	240
HANChild4_UL82	AIIRRTAHYWAVRLTVTPNWRRRTDSSLEAGQIFVSQFAFRAGAIPLTLVDALEQLACSDPNTYIHKTETDERGQWIMLF	240
HANRTR2_UL82	AIIRRTAHHWAVRLTVTPNWRRRTDSSLEAGQIFVSQFAFRAGAIPLTLVDALEQLACSDPNTYIHKTETDERGQWIMLF	240
HANRTR4_UL82	AIIRRTAHHWAVRLTVTPNWRRRTDSSLEAGQIFVSQFAFRAGAIPLTLVDALEQLACSDPNTYIHKTETDERGQWIMLF	240
HANRTR5_UL82	AIIRRTAHHWAVRLTVTPNWRRRTDSSLEAGQIFVSQFAFRAGAIPLTLVDALEQLACSDPNTYIHKTETDERGQWIMLF	240
HANSCTR1B_UL82	AIIRRTAHHWAVRLTVTPNWRRRTDSSLEAGQIFVSQFAFRAGAIPLTLVDALEQLACSDPNTYIHKTETDERGQWIMLF	240
HANSCTR4_UL82	AIIRRTAHHWAVRLTVTPNWRRRTDSSLEAGQIFVSQFAFRAGAIPLTLVDALEQLACSDPNTYIHKTETDERGQWIMLF	240
UKLon1Blood2013_UL82	AIIRRTAHYWAVRLTVTPNWRRRTDSSLEAGQIFVSQFAFRAGAIPLTLVDALEQLACSDPNTYIHKTETDERGQWIMLF	240
UKLon6Urine2011_UL82	AIIRRTAHYWAVRLTVTPNWRRRTDSSLEAGQIFVSQFAFRAGAIPLTLVDALEQLACSDPNTYIHKTETDERGQWIMLF	240
PAV1_UL82	AIIRRTAHHWAVRLTVTPNWRRRTDSSLEAGQIFVSQFAFRAGAIPLTLVDALEQLACSDPNTYIHKTETDERGQWIMLF	240
Lab_AD169_UL82	AIIRRTAHHWAVRLTVTPNWRRRTDSSLEAGQIFVSQFAFRAGAIPLTLVDALEQLACSDPNTYIHKTETDERGQWIMLF	240
Lab_FIX-UL82	AIIRRTAHHWAVRLTVTPNWRRRTDSSLEAGQIFVSQFAFRAGAIPLTLVDALEQLACSDPNTYIHKTETDERGQWIMLF	240
Lab_Merlin-UL82	AIIRRTAHHWAVRLTVTPNWRRRTDSSLEAGQIFVSQFAFRAGAIPLTLVDALEQLACSDPNTYIHKTETDERGQWIMLF	240
Lab_TB40e-UL82	AIIRRTAHYWAVRLTVTPNWRRRTDSSLEAGQIFVSQFAFRAGAIPLTLVDALEQLACSDPNTYIHKTETDERGQWIMLF	240
Lab_Towne_UL82	AIIRRTAHYWAVRLTVTPNWRRRTDSSLEAGQIFVSQFAFRAGAIPLTLVDALEQLACSDPNTYIHKTETDERGQWIMLF	240
Lab_TR_UL82	AIIRRTAHHWAVRLTVTPNWRRRTDSSLEAGQIFVSQFAFRAGAIPLTLVDALEQLACSDPNTYIHKTETDERGQWIMLF	240
Lab_Herberling-UL82	VDIRRTAYHWGVRTTVSPRWRRKVDRSLEAEQIFTTEFIFRAGAIPLRLVDAVELLSCSDRNTYIHKAETDARGQWVNVH	237

**Consensus**	**LHHDSPHPPTSVFLHFSVYTHRAEVVARHNPYPHLRRLPDNGFQLLIPKSFTLTRIHPEYIVQIQNAFETNQTHDTIFFP**	
	250 260 270 280 290 300 310 320	
BE212010_UL82	LHHDSPHPPTSVFLHFSVYTHRAEVVARHNPYPHLRRLPDNGFQLLIPKSFTLTRIHPEYIVQIQNAFETNQTHDTIFFP	320
HANChild4_UL82	LHHDSPHPPTSVFLHFSVYTHRAEVVARHNPYPHLRRLPDNGFQLLIPKSFTLTRIHPEYIVQIQNAFETNQTHDTIFFP	320
HANRTR2_UL82	LHHDSPHPPTSVFLHFSVYTHRAEVVARHNPYPHLRRLPDNGFQLLIPKSFTLTRIHPEYIVQIQNAFETNQTHDTIFFP	320
HANRTR4_UL82	LHHDSPHPPTSVFLHFSVYTHRAEVVARHNPYPHLRRLPDNGFQLLIPKSFTLTRIHPEYIVQIQNAFETNQTHDTIFFP	320
HANRTR5_UL82	LHHDSPHPPTSVFLHFSVYTHRAEVVARHNPYPHLRRLPDNGFQLLIPKSFTLTRIHPEYIVQIQNAFETNQTHNTIFFP	320
HANSCTR1B_UL82	LHHDSPHPPTSVFLHFSVYTHRAEVVARHNPYPHLRRLPDNGFQLLIPKSFTLTRIHPEYIVQIQNAFETNQTHDTIFFP	320
HANSCTR4_UL82	LHHDSPHPPTSVFLHFSVYTHRAEVVARHNPYPHLRRLPDNGFQLLIPKSFTLTRIHPEYIVQIQNAFETNQTHDTIFFP	320
UKLon1Blood2013_UL82	LHHDSPHPPTSVFLHFSVYTHRAEVVARHNPYPHLRRLPDNGFQLLIPKSFTLTRIHPEYIVQIQNAFETNQTHDTIFFP	320
UKLon6Urine2011_UL82	LHHDSPHPPTSVFLHFSVYTHRAEVVARHNPYPHLRRLPDNGFQLLIPKSFTLTRIHPEYIVQIQNAFETNQTHDTIFFP	320
PAV1_UL82	LHHDSPHPPTSVFLHFSVYTHRAEVVARHNPYPHLRRLPDNGFQLLIPKSFTLTRIHPEYIVQIQNAFETNQTHDTIFFP	320
Lab_AD169_UL82	LHHDSPHPPTSVFLHFSVYTHRAEVVARHNPYPHLRRLPDNGFQLLIPKSFTLTRIHPEYIVQIQNAFETNQTHDTIFFP	320
Lab_FIX-UL82	LHHDSPHPPTSVFLHFSVYTHRAEVVARHNPYPHLRRLPDNGFQLLIPKSFTLTRIHPEYIVQIQNAFETNQTHDTIFFP	320
Lab_Merlin-UL82	LHHDSPHPPTSVFLHFSVYTHRAEVVARHNPYPHLRRLPDNGFQLLIPKSFTLTRIHPEYIVQIQNAFETNQTHDTIFFP	320
Lab_TB40e-UL82	LHHDSPHPPTSVFLHFSVYTHRAEVVARHNPYPHLRRLPDNGFQLLIPKSFTLTRIHPEYIVQIQNAFETNQTHDTIFFP	320
Lab_Towne_UL82	LHHDSPHPPTSVFLHFSVYTHRAEVVARHNPYPHLRRLPDNGFQLLIPKSFTLTRIHPEYIVQIQNAFETNQTHDTIFFP	320
Lab_TR_UL82	LHHDSPHPPTSVFLHFSVYTHRAEVVARHNPYPHLRRLPDNGFQLLIPKSFTLTRIHPEYIVQIQNAFETNQTHDTIFFP	320
Lab_Herberling-UL82	LQHETLHPPPSVFLHFSLYTHGAEVVLRHNPYPHLTRHGDNGFTLHAPRGFTLSRLHREYIVQVQNAFETNNTHDVIFFP	317

```
Consensus              ENIPGVSIEAGPLPDRVRITLRVTLTGDQAVHLEHRQPLGRIHFFRRGFWTLTPGKPDKIKRPQVQLRAGLFPRSNVMRG
                                330       340       350       360       370       380       390       400
BE212010_UL82          ENIPGVSIEAGPLPDRVRITLRVTLTGDQAVHLEYRQPLGRIHFFRRGFWTLTPGKPDKIKRPQVQLRAGLFPRSNVMRG  400
HANChild4_UL82         ENIPGVSIEAGPLPDRVRITLRVTLTGDQAVHLEHRQPLGRIHFFRRGFWTLTPGKPDKIKRPQVQLRAGLFPRSNVMRG  400
HANRTR2_UL82           ENIPGVSIEAGPLPDRVRITLRVTLTGDQAVHLEHRQPLGRIHFFRRGFWTLTPGKPDKIKRPQVQLRAGLFPRSNVMRG  400
HANRTR4_UL82           ENIPGVSIEAGPLPDRVRITLRVTLTGDQAVHLEHRQPLGRIHFFRRGFWTLTPGKPDKIKRPQVQLRAGLFPRSNVMRG  400
HANRTR5_UL82           ENIPGVSIEAGPLPDRVRITLRVTLTGDQAVHLEHRQPLGRIHFFRRGFWTLTPGKPDKIKRPQVQLRAGLFPRSNVMRG  400
HANSCTR1B_UL82         ENIPGVSIEAGPLPDRVRITLRVTLTGDQAVHLEHRQPLGRIHFFRRGFWTLTPGKPDKIKRPQVQLRAGLFPRSNVMRG  400
HANSCTR4_UL82          ENIPGVSIEAGPLPDRVRITLRVTLTGDQAVHLEHRQPLGRIHFFRRGFWTLTPGKPDKIKRPQVQLRAGLFPRSNVMRG  400
UKLon1Blood2013_UL82   ENIPGVSIEAGPLPDRVRITLRVTLTGDQAVHLEHRQPLGRIHFFRRGFWTLTPGKPDKIKRPQVQLRAGLFPRSNVMRG  400
UKLon6Urine2011_UL82   ENIPGVSIEAGPLPDRVRITLRVTLTGDQAVHLEHRQPLGRIHFFRRGFWTLTPGKPDKIKRPQVQLRAGLFPRSNVMRG  400
PAV1_UL82              ENIPGVSIEAGPLPDRVRITLRVTLTGDQAVHLEHRQPLGRIHFFRRGFWTLTPGKPDKIKRPQVQLRAGLFPRSNVMRG  400
Lab_AD169_UL82         ENIPGVSIEAGPLPDRVRITLRVTLTGDQAVHLEHRQPLGRIHFFRRGFWTLTPGKPDKIKRPQVQLRAGLFPRSNVMRG  400
Lab_FIX-UL82           ENIPGVSIEAGPLPDRVRITLRVTLTGDQAVHLEHRQPLGRIHFFRRGFWTLTPGKPDKIKRPQVQLRAGLFPRSNVMRG  400
Lab_Merlin-UL82        ENIPGVSIEAGPLPDRVRITLRVTLTGDQAVHLEHRQPLGRIHFFRRGFWTLTPGKPDKIKRPQVQLRAGLFPRSNVMRG  400
Lab_TB40e-UL82         ENIPGVSIEAGPLPDRVRITLRVTLTGDQAVHLEHRQPLGRIHFFRRGFWTLTPGKPDKIKRPQVQLRAGLFPRSNVMRG  400
Lab_Towne_UL82         ENIPGVSIEAGPLPDRVRITLRVTLTGDQAVHLEHRQPLGRIHFFRRGFWTLTPGKPDKIKRPQVQLRAGLFPRSNVMRG  400
Lab_TR_UL82            ENIPGVSIEAGPLPDRVRITLRVTLTGDQAVHLEHRQPLGRIHFFRRGFWTLTPGKPDKIKRPQVQLRAGLFPRSDVVRG  400
Lab_Herberling-UL82    ADIPGVSMEAGPLPDRVRITIRLTWTGENSVRIEHMQILGTIHLFKRGVLNLLPGKTEKIKRPQIQLRAGLFPRRAVMRG  397

Consensus              AVSEFLPQSPGLPPTEEEEEEEEEEDDEDDLSSTPTPTPLSEAMFAGFEEASGDEDSDTQAGLSRALILTGQR--RRSGNN
                                410       420       430       440       450       460       470       480
BE212010_UL82          AVSEFLPQSPGLPPTEEEEEEEEEEDDEDDLSSTPTPTPLSEAMFAGFEEASGDEDSDTQAGLSRALILTGQR--RRSGNN  478
HANChild4_UL82         AVSEFLPQSPGLPPTEEEEEEEEEEDDEDDLSSTPTPTPLSEAMFAGFEEASGDEDSDTQAGLSRALILTGQR--RRSGNN  478
HANRTR2_UL82           AVSEFLPQSPGLPPTEEEEEEEEEEDDEDDLSSTPTPTPLSEAMFAGFEEASGDEDSDTQAGLSRALILTGQR--RRSGNN  478
HANRTR4_UL82           AVSEFLPQSPGLPPTEEEEEEEEEEDDEDDLSSTPTPTPLSEAMFAGFEEASGDEDSDTQAGLSRALILTGQR--RRSGNN  478
HANRTR5_UL82           AVSEFLPQSPGLPPTEEEEEEEEEEDDEDDLSSTPTPTPLSEAMFAGFEEASGDEDSDTQAGLSRALILTGQR--RRSGNN  478
HANSCTR1B_UL82         AVSEFLPQSPGLPPTEEEEEEEEEEDDEDDLSSTPTPTPLSEAMFAGFEEASGDEDSDTQAGLSRALILTGQR--RRSGNN  478
HANSCTR4_UL82          AVSEFLPQSPGLPPTEEEEEEEEEEDDEDDLSSTPTPTPLSEAMFAGFEEASGDEDSDTQAGLSRALILTGQR--RRSGNN  478
UKLon1Blood2013_UL82   AVSEFLPQSPGLPPTEEEEEEEEEEDDEDDLSSTPTPTPLSEAMFAGFEEASGDEDSDTQAGLSRALILTGQR--RRSGNN  478
UKLon6Urine2011_UL82   AVSEFLPQSPGLPPTEEEEEEEEEEDDEDDLSSTPTPTPLSEAMFAGFEEASGDEDSDTQAGLSRALILTGQR--RRSGNN  478
PAV1_UL82              AVSEFLPQSPGLPPTEEEEEEEEEEDDEDDLSSTPTPTPLSEAMFAGFEEASGDEDSDTQAGLSRALILTGQR--RRSGNN  478
Lab_AD169_UL82         AVSEFLPQSPGLPPTEEEEEEEEEEDDEDDLSSTPTPTPLSEAMFAGFEEASGDEDSDTQAGLSRALILTGQR--RRSGNN  478
Lab_FIX-UL82           AVSEFLPQSPGLPPTEEEEEEEEEEDDEDDLSSTPTPTPLSEAMFAGFEEASGDEDSDTQAGLSRALILTGQR--RRSGNN  478
Lab_Merlin-UL82        AVSEFLPQSPGLPPTEEEEEEEEEEDDEDDLSSTPTPTPLSEAMFAGFEEASGDEDSDTQAGLSRALILTGQR--RRSGNN  478
Lab_TB40e-UL82         AVSEFLPQSPGLPPTEEEEEEEEEEDDEDDLSSTPTPTPLSEAMFAGFEEASGDEDSDTQAGLSRALILTGQR--RRSGNN  478
Lab_Towne_UL82         AVSEFLPQSPGLPPTEEEEEEEEEEDDEDDLSSTPTPTPLSEAMFAGFEEASGDEDSDTQAGLSRALILTGQR--RRSGNN  478
Lab_TR_UL82            AVSEFLPQSPGLPPTEEEEEEEEEEDDEDDLSSTPTPTPLSEAMFAGFEEASGDEDSDTQAGLSRALILTGQR--RRSGNN  478
Lab_Herberling-UL82    EVSEFRPQSPGELPLEGEEEEEEEEER---SSTPTPPALSESVFAAFEESSEEEESDTEEGLSRALALTGRRRPRRGADE  474
```

**Consensus**	**GA-LTLVIPSWHVFASLDDLVPLTVSVQHAALRPTSYLRSDMDGDVRTAADISSTLRSVPAPRPSPISTASTSST--PRS**	
	490 500 510 520 530 540 550 560	
BE212010_UL82	GA-LTLVIPSWHVFASLDDLVPLTVSVQHAALRPTSYLRSDMDGDVRTAADISSTLRSVPAPRPSPISTASTSST--PRS	555
HANChild4_UL82	GA-LTLVIPSWHVFASLDDLVPLTVSVQHAALRPTSYLRSDMDGDVRTAADISSTLRSVPAPRPSPISTASTSST--PRS	555
HANRTR2_UL82	GA-LTLVIPSWHVFASLDDLVPLTVSVQHAALRPTSYLRSDMDGDVRTAADISSTLRSVPAPRPSPISTASTSST--PRS	555
HANRTR4_UL82	GA-LTLVIPSWHVFASLDDLVPLTVSVQHAALRPTSYLRSDMDGDVRTAADISSTLRSVPAPRPSPISTASTSST--PRS	555
HANRTR5_UL82	GA-LTLVIPSWHVFASLDDLVPLTVSVQHAALRPTSYLRSDMDGDVRTAADISSTLRSVPAPRPSPISTASTSST--PRS	555
HANSCTR1B_UL82	GA-LTLVIPSWHVFASLDDLVPLTVSVQHAALRPTSYLRSDMDGDVRTAADISSTLRSVPAPRPSPISTASTSST--PRS	555
HANSCTR4_UL82	GA-LTLVIPSWHVFASLDDLVPLTVSVQHAALRPTSYLRSDMDGDVRTAADISSTLRSVPAPRPSPISTASTSST--PRS	555
UKLon1Blood2013_UL82	GA-LTLVIPSWHVFASLDDLVPLTVSVQHGALRPTSYLRSDMDGDVRTAADISSTLRSVPAPRPSPISTASTSST--PRS	555
UKLon6Urine2011_UL82	GA-LTLVIPSWHVFASLDDLVPLTVSVQHGALRPTSYLRSDMDGDVRTAADISSTLRSVPAPRPSPISTASTSST--PRS	555
PAV1_UL82	GA-LTLVIPSWHVFASLDDLVPLTVSVQHAALRPTSYLRSDMDGDVRTAADISSTLRSVPAPRPSPISTASTSST--PRS	555
Lab_AD169_UL82	GA-LTLVIPSWHVFASLDDLVPLTVSVQHAALRPTSYLRSDMDGAVRTAADISSTLRSVPAPRPSPISTASTSST--PRS	555
Lab_FIX-UL82	GA-LTLVIPSWHVFASLDDLVPLTVSVQHAALRPTSYLRSDMDGDVRTAADISSTLRSVPAPRPSPISTASTSST--PRS	555
Lab_Merlin-UL82	GA-LTLVIPSWHVFASLDDLVPLTVSVQHAALRPTSYLRSDMDGDVRTAADISSTLRSVPAPRPSPISTASTSST--PRS	555
Lab_TB40e-UL82	GA-LTLVIPSWHVFASLDDLVPLTVSVQHAALRPTSYLRSDMDGDVRTAADISSTLRSVPAPRPSPISTASTSST--PRS	555
Lab_Towne_UL82	GA-LTLVIPSWHVFASLDDLVPLTVSVQHAALRPTSYLRSDMDGDVRTAADISSTLRSVPAPRPSPISTASTSST--PRS	555
Lab_TR_UL82	GA-LTLVIPSWHVFASLDDLVPLTVSVQHAALRPTSYLRSDMDGDVRTAADISSTLRSVPAPRPSPISTASTSST--PRS	555
Lab_Herberling-UL82	GEDLMLVIPSWNVFVNIDNLVPLTGSVEQAALKPTSYLRSEMQGDVRTAADFTSNLQPVPVPRPSPMSLPSTSGTAASRS	554

**Consensus**	**RPRI**	
BE212010_UL82	RPRI	559
HANChild4_UL82	RPRI	559
HANRTR2_UL82	RPRI	559
HANRTR4_UL82	RPRI	559
HANRTR5_UL82	RPRI	559
HANSCTR1B_UL82	RPRI	559
HANSCTR4_UL82	RPRI	559
UKLon1Blood2013_UL82	RPRI	559
UKLon6Urine2011_UL82	RPRI	559
PAV1_UL82	RPRI	559
Lab_AD169_UL82	RPRI	559
Lab_FIX-UL82	RPRI	559
Lab_Merlin-UL82	RPRI	559
Lab_TB40e-UL82	RPRI	559
Lab_Towne_UL82	RPRI	559
Lab_TR_UL82	RPRI	559
Lab_Herberling-UL82	RPRI	558

Consensus Threshold: >80%

Compare to: the consensus

Amino acids that don't match the reference are marked with pink highlighting.

Created: Dec 28, 2023

Last Modified: Dec 28, 2023
