# Supplementary figures and images for: Cytotoxic lymphocytes counteract viral type I interferon immune evasion
Source: PLoS Pathog. 2026 Feb 9;22(2):e1013955. doi: 10.1371/journal.ppat.1013955 (PMC12912695; doi:10.1371/journal.ppat.1013955)

S2 Fig

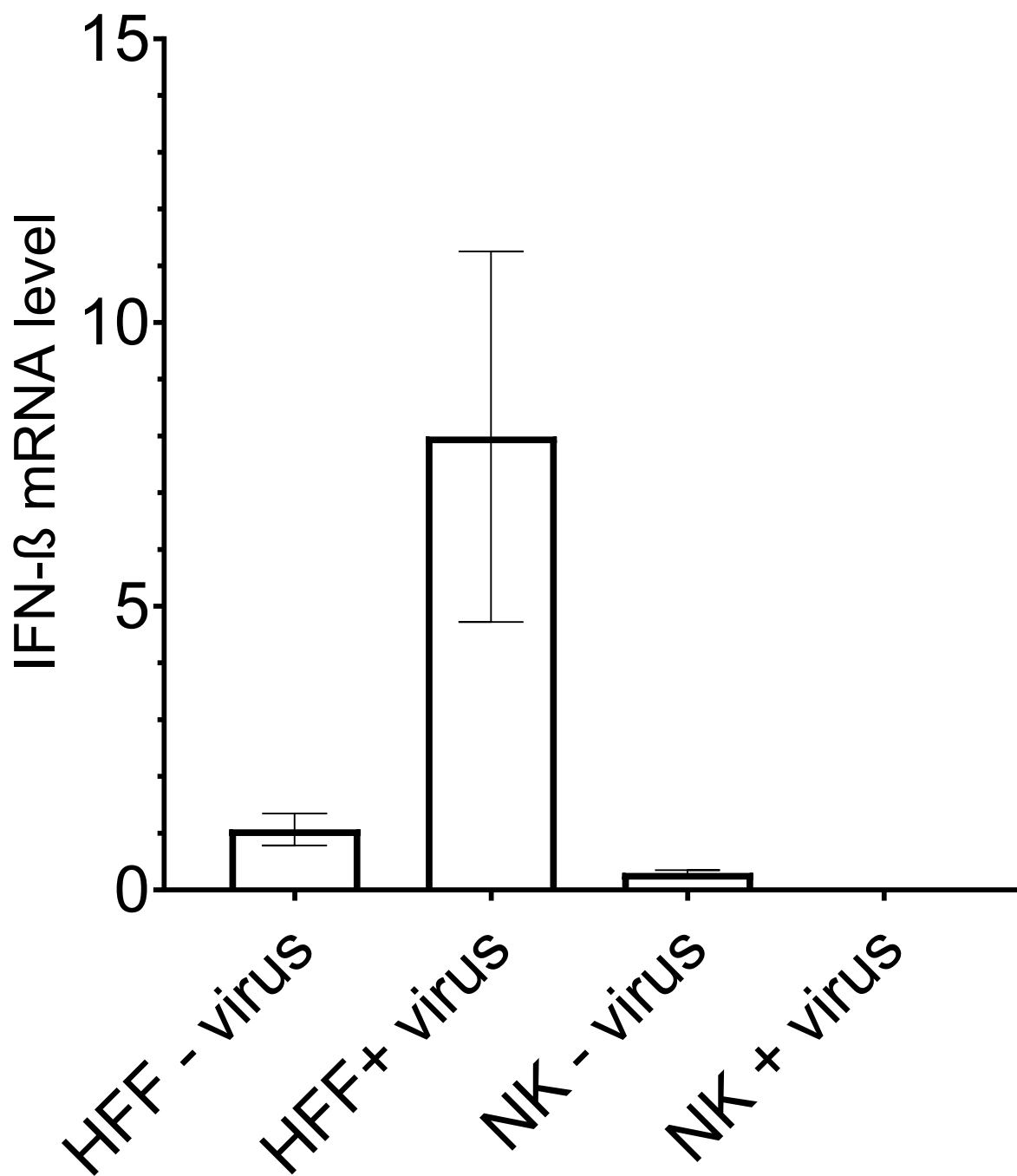

Supplement: S2 Fig — HFF or NK cells were incubated with or without HCMV-AD169 (MOI = 0.2) for 2 hours and cultured overnight. Then, mRNA for IFN-β was measured by qPCR with HFF only (without virus) set at 1. (PDF) [file ppat.1013955.s002.pdf]
