# Supplementary material for: Cytotoxic lymphocytes counteract viral type I interferon immune evasion
Source: PLoS Pathog. 2026 Feb 9;22(2):e1013955. doi: 10.1371/journal.ppat.1013955 (PMC12912695; doi:10.1371/journal.ppat.1013955)
Supplement: S3 Fig — All data used to make the Figs, also including full immunoblots. (PDF) [file ppat.1013955.s003.pdf]

Sup Fig. 3

Fig 1a

Virus type	Infection status	Mean	SEM	n (triplicates)
AD169	-	1,0475	0,06	6
AD169	+	7,99	1,092	3
Merlin	-	1,013	0,108	3
Merlin	+	0,013	0,004	3

Fig. 1b

	0:1			1:1			2:1			4:1		
	Mean	SEM	n (triplicates)	Mean	SEM	n (triplicates)	Mean	SEM	n (triplicates)	Mean	SEM	n (triplicates)
Not Infected	1,0475	0,06	6	1,426	0,194	3	1,919	0,293	3	0,87	0,075	3
Infected	7,99	1,092	3	22,648	1,344	3	29,184	0,558	3	36,502	2,14	3
Infected normalized to 0:1	1	1	3	2,834543179	0,168210263	3	3,652565707	0,069837297	3	4,568460576	0,267834793	3

Fig. 1c

	0:1			1:1			2:1			4:1		
	Mean	SEM	n (triplicates)	Mean	SEM	n (triplicates)	Mean	SEM	n (triplicates)	Mean	SEM	n (triplicates)
Not Infected	1,547	0,357	3	15,627	3,966	3	16,27	4,427	3	12,618	2,088	3
Merlin Infected	0,757	0,18	3	370,075	105,274	3	442,371	112,921	3	552,563	122,342	3

Fig. 1d

p-IRF3

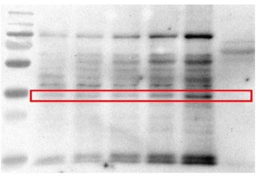

Control

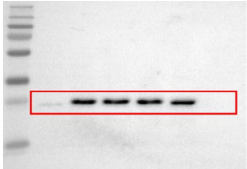

Fig. 1e

	0:1			1:1			2:1		
	Mean	SD	n (triplicates)	Mean	SD	n (triplicates)	Mean	SD	n (triplicates)
Not Infected	1,6	2,24	4	2,83	3,21	3	5,81	2,74	3
Infected	17,55	7,93	4	48,93	8,25	3	52,86	11,12	3

Fig. 1f

	0:1	0.1:1	0.3:1
Not Infected	1,310797	0,115143	0,048261
	0,811901	0,825306	0,06346
	0,93964	0,088388	0,075389
	7,507252	1,850744	1,654358
	0,351571	2,046485	2,286398
	0,378884	0,96957	2,557911
		8,159486	19,30911
		0,838132	0,986309
		0,497532	0,427355

	0:1	0.1:1	0.3:1
AD169	1,070907	7,691864	7,489313
	0,971532	7,246744	7,264346
	0,96115	6,93951	35,674797
	0,88693	10,222717	19,672815
	0,764421	9,932108	29,584683
	1,474953	30,065361	24,106316
	2,66626	52,666519	46,696901
	0,58268	18,527735	36,749833
	0,643676		58,743515

Fig. 1g

	0:1	0.1:1	0.3:1
Not Infected	0,31630365	1,55449711	2,343176
	1,34959559	2,26798599	1,21679598
	1,87004724	0,44260917	1,5809628
	1,25267833	17,54865855	1,00684793
	1,287072	0,685196	2,801444
	0,904589	0,951274	0,914026
	0,992516	2,085742	0,481705
	0,865383	3,678147	1,707872

	0:1	0.1:1	0.3:1
AD169	1,74027752	3,54296115	3,95209124
	0,69501803	4,87229785	6,14986728
	0,90301618	3,3809862	4,77317641
	0,91556649	2,91655825	5,61650369
	1,331732	10,293545	11,415621
	1,330808	13,283217	1,872586
	0,6752	4,511358	4,446369
	0,835671	3,625186	5,355559
	1,005092	8,737095	12,531599
	1,518068	9,351672	12,07047
	0,655395	7,031457	21,352899
	1,723944	17,02498	23,176264
	0,716991	18,286169	31,378941
	0,809027	16,775926	20,356572

Fig. 1h

	pp71 410ng		
	Mean	SD	n (triplicates)
pp71 0 ng	100,00	5,59	3
0:1	30,01	10,44	3
1:1	68,04	6,69	3
2:1	76,99	2,27	3
4:1	87,06	5,39	3
8:1	98,97	7,78	3
16:1	109,38	7,19	3

pp71

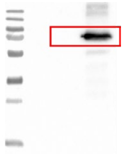

GAPDH

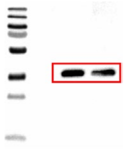

Fig. 1i

pp71 GAPDH

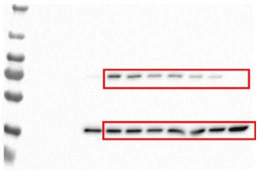

Fig. 2a

Days	WT										Mutant									
-0.3	12832554	9262169	7050413	10030405	8335680	5544927	6657559.5	6714421	5414587.5	6824568.5	35270228	12271607	11692538	12923670	17208750	8223130	11978367	8676659	11039003	12651503
0	28454.258	38612.512	89319.344	43956.117	92214.313	36987.047	18558.346	72941.953	48870.086	117267.773	126549.211	187000.344	133104.859	108206.383	235107.219	147669.109	348566.438	139558.203	145488.781	189387.75
1	38545.719	46337.227	56793.309	113905.352	53838.387	60829.535	61132.781	195888.813	69338.641	102616.719	1376497.5	968475.125	995907.813	1276447.25	1059455.125	989356.688	942937.313	859376.813	1040775.25	983046
2	93901.859	107016.93	57566.109	70233.133	49604.043	99453.781	124760.258	86440.5	104444.188	75791.07	845081.313	1385504.5	779777.938	665453.625	901284.5	624486.938	777859.625	842603.813	750484.813	1059219.25
3	1420936.25	734722.188	676966.813	369338.906	621156.875	1179856.375	656354.5	599529.875	400279.25	435731.156	270978.906	454010.813	464220.031	600805.563	465077.188	392514.313	590071.5	5895022.5	618760.25	625046.438
6	31861906	42670892	46288136	29470296	32043732	20177480	23812924	28409124	22721000	26112992	49160312	32317928	26849138	36713224	53340508	36914368	19375228	20868940	19646212	22180244
7	31449428	38374396	47247316	48424084	45868760	33439572	37353804	32128168	26371706	29435512	96373256	62138332	56936748	54476292	89184592	55689832	38302104	41186980	36117880	33101712

Fig. 2b

	pp71 L439A ISRE								
0	112.9707	95.58238	91.44695	107.3293	97.40926	95.2614	128.3491	105.3273	66.32366
10	64.63435	63.75812	76.76843	52.44779	49.29115	53.17704	58.46208	60.15103	65.88896
50	107.4399	80.6431	92.5059	65.64748	55.89961	56.10573	89.17902	78.85097	83.51415

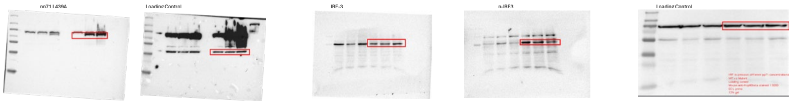

Fig. 2c

23.4.2018

	pp71 FL ISRE								
0	103.9396	96.70624	99.35418	98.92556	97.98669	103.0877	94.97532	100.918	104.1066
10	58.81563	63.97824	71.22509	39.70519	55.548	58.55532	53.44572	60.90098	65.25976
50	71.22676	64.49927	48.35454	57.48343	48.52166	48.93515	69.01371	56.29696	57.06164

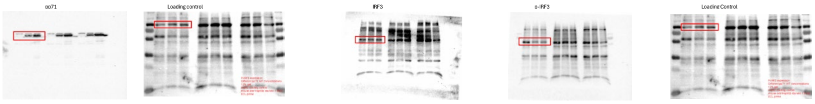

Fig. 2d

	0:1			0.5:1			1:1			2:1			4:1		
	Mean	SEM	n (triplicates)	Mean	SEM	n (triplicates)	Mean	SEM	n (triplicates)	Mean	SEM	n (triplicates)	Mean	SEM	n (triplicates)
WT Infected	1.115	0.271	3	12.869	1.513	3	18.807	0.534	3	20.576	2.84	3	35.623	2.182	3
Mutant Infected	1.031	0.156	3	4.14	0.195	3	10.743	2.401	3	6.809	1.63	3	5.901	0.899	3

Fig. 2e Flow cytometry data in manuscript

Fig. 2f

	HCMV-HFFs + WT-NK									HCMV-HFFs + GrM-KO-NK								
HFFs	1.087991	0.938655	0.979194	0.8928	1.61926	0.691755	1.105	0.963708	0.938654	1.087991	0.938655	0.979194	0.8928	1.61926	0.691755	1.105475	0.963708	0.938654397
0:1	7.898978	8.366743	6.053062	5.681738	7.208	10.2983	11.086	10.481		7.233365	14.25766	5.957332	5.681738	7.208344026	10.2983	11.08599	10.48073	
1:1	28.3773	41.80679	25.06609	19.39124	21.78609	26.76609	38.899	35.919		17.89725	38.204	17.1801	22.10551	46.47342	23.4633	31.993		
2:1	35.03357	30.58315	38.55018	29.39163	27.69079	30.47036	50.202	25.381	20.417	21.62557	22.85863	22.68499	28.41016	19.55321	28.17482	19.817	17.038	
4:1	23.84584	32.73281	33.74651	30.13428	39.02517	40.34543	39.937	23.665		20.71589	13.0291	21.4613	28.5681	14.0095	27.67157	21.746	24.858	

Fig. 2g

HFFs	HCMV-HFFs + WT-NK										HCMV-HFFs + GrM-KO-NK								
4:1	104.9	95.1	79.2	108.7	112.1	82.6	106.9	110.5	125.6	74.4	58.4	61.7	61.3	77.8	53.6	77.2	62.3	53.6	

Fig. 2h

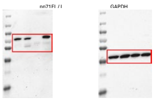

Fig. 2i Flow cytometry data in manuscript

Fig. 2j

WT NK	138.63	91.66	78.65	91.06	94.96	163.16	71	70.88	109.86	89.71	80.36	120.07	110.15	86.5	103.35
Perforin-KO NK	55.67	35.89	32.05	28.53	44.52	56.69	23.82	65.08	39.89	66.84	58.02	44.11	72.88		

Fig. 2k

	- decoy proteins															+ decoy proteins														
WT NK	138.63	91.66	78.65	91.06	94.96	163.16	71	70.88	109.86	89.71	80.36	120.07	110.15	86.5	103.35	92.45	48.13	66.95	54.1	20.13	52.8	17.23	108.21	95.18	104.27	52.78	134.24	59.4		
Perforin-KO NK	55.67	35.89	32.05	28.53	44.52	56.69	23.82	65.08	39.89	66.84	58.02	44.11	72.88			23.19	24.15	24.79	45.72	13.16	11.26	17.51	33.85	46.93	26.49	65.04	72.18	67.42	37.98	28.38

Fig. 3a-b In manuscript

Fig. 3c

	% ISRE promoter activation		
	**Mean**	**SD**	**n (triplicates)**
**EV**	0,80	0,05	3
**cGAS**	1,39	0,17	3
**STING**	2,14	0,17	3
**cGAS + STING**	100,00	16,21	3

Fig. 3d

	pp71 FL								
**0**	105,8283	93,38776	100,784	97,2216	103,17	99,60839	95,97967	98,09023	105,9301
**10**	69,90392	65,08447	69,99403	91,85533	113,8762	86,53097	66,26706	60,78049	66,93234
**50**	70,84819	72,03282	69,91062	69,25708	77,7396	76,11493	57,47085	51,78793	52,1685
**410**	18,97019	21,03524	21,29335	24,91732	19,68176	18,00808	19,8657	16,99231	18,17068

pp71FL

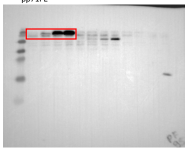

Fig. 3e

	pp71 L								
**0**	106,9285	107,4243	85,64719	104,698	95,99482	99,30719	102,8825	99,38523	97,73231
**10**	121,2233	123,9054	108,2145	124,6771	104,1082	156,8309	94,3513	92,43209	89,87556
**50**	166,7627	155,6625	165,499	128,8126	125,106	153,2678	148,8367	148,6032	138,6236
**410**	134,4497	113,4327	90,30098	162,0788	173,1798	140,0021	171,5677	152,6474	153,7783

pp71L

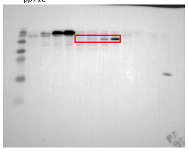

Fig. 3f

	pp71 S								
**0**	106,0709	92,42733	101,5017	105,5049	104,9291	89,56609	89,76859	127,5895	82,64193
**10**	105,2609	93,61728	102,0389	103,5873	95,48111	118,0948	105,7062	99,07629	83,66158
**50**	95,04783	93,43652	86,33906	96,66823	79,34992	85,71204	81,92102	80,42046	75,27212
**410**	84,79054	89,20821	81,98665	85,08208	80,76574	66,85438	82,45567	76,73796	80,78792

pp71S

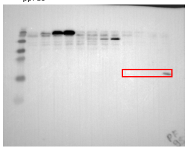

Fig. 3g

	% IFNB promoter activation		
	**Mean**	**SD**	**n (triplicates)**
**EV**	4,81	2,05	3
**cGAS**	6,56	0,28	3
**STING**	9,27	0,57	3
**cGAS + STING**	100,00	6,42	3

Fig. 3h

	pp71 FL								
**0**	133,1826	82,63744	84,17999	95,6365	111,5135	92,85001	99,00612	95,37936	105,6145
**10**	63,18532	66,29363	69,75855	40,4729	39,36017	36,49821	31,12987	27,7938	26,14126
**50**	66,20489	68,20901	71,86443	51,17392	48,69605	44,52055	68,17144	45,64681	63,22773
**410**	40,03123	45,93954	53,53191	14,88095	26,10387	38,88068	44,36382	29,96746	50,0028

pp71FL

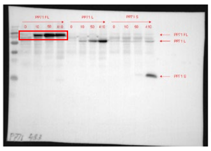

Fig. 3i

	pp71 L								
**0**	96,43334	101,6219	101,9448	82,72167	101,7622	115,5161	90,27614	103,1126	106,6113
**10**	273,4034	251,8536	256,814	156,2691	172,3408	205,0245	130,3332	140,1035	138,7801
**50**	524,3549	447,0218	343,0918	250,0954	306,151	303,8152	220,6102	260,6012	220,9974
**410**	346,0102	347,608	363,3771	248,1709	288,6614	244,0701	228,6776	231,5543	227,97

pp71L

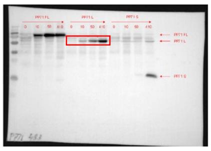

Fig. 3j

	pp71 S								
**0**	120,6229	98,5601	80,81698	109,611	88,55598	101,833	110,203	85,92608	103,8709
**10**	50,60695	65,90621	88,96146	81,96966	77,36062	75,65798	47,49875	48,9455	45,53299
**50**	100,498	96,68839	93,56092	64,95804	48,71488	47,1754	102,7002	88,95024	107,3469
**410**	26,03811	30,54622	33,87462	67,49087	19,66472	23,47363	46,48961	47,79216	45,49624

pp71S

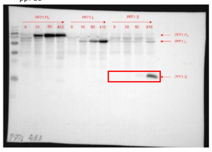

Fig. 3k

	pp71 L + S								
**0**	92,88208	79,05777	76,91637	103,5188	96,02801	103,0059	116,258	112,8536	119,4794
**10**	76,63502	74,45982	77,58827	104,2097	99,85766	111,1781	109,3692	109,9469	101,1369
**50**	78,46394	71,24214	68,71967	94,01496	103,848	101,1334	72,13812	96,93664	84,13451
**410**	14,8573	15,97986	12,27041	24,52783	22,06362	23,19517	35,65287	29,39497	35,32824

pp71L pp71S

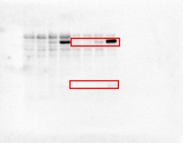

Fig. 4a

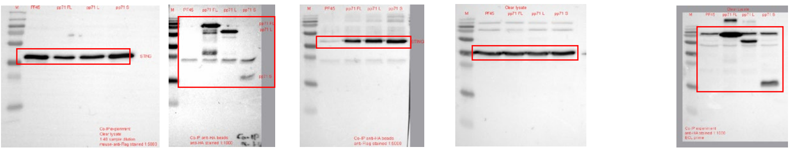

Fig. 4b

	pp71 FL + L %					
**No pp71 FL**	103,1749	101,5572	95,26792			
**0**	38,11082	39,31729	40,0585	42,91998	48,92958	44,96734
**25**	53,23758	52,38864	49,54032	78,1653	67,55409	73,93979
**75**	91,29376	86,52515	93,53846	84,43538	78,73936	80,72233
**100**	91,8056	83,86271	86,38756	73,15833	73,59913	60,02213
**205**	86,68674	71,96053	85,43987	71,01377	75,08961	67,93453

pp71FL pp71L

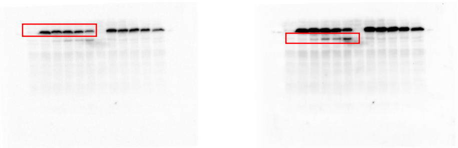

Fig. 4c

	pp71 FL + S					
**No pp71 FL**	102,3203	94,78173	102,898			
**0**	46,38429	46,69714	46,60233	42,41248	43,95435	40,85177
**25**	35,09196	32,80502	37,40777	37,37195	38,21199	41,85797
**75**	30,95045	33,84827	30,12153	33,65262	30,87926	29,00971
**100**	33,84225	35,74082	38,0639	21,9449	21,88545	24,93352
**205**	15,12747	15,27	15,36698	13,92841	10,87663	13,80194

pp71FL pp71S

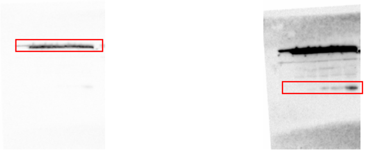

Fig. 4d

	pp71 FL + L + S					
**No pp71 FL**	122,0032	107,3508	70,64599			
**0**	52,7655	65,18796	71,71179	50,74517	48,39781	46,25438
**25**	75,34094	77,91995	74,9451	57,02487	55,44535	58,51809
**75**	43,79006	44,04351	43,12896	31,4748	31,08441	29,27392
**100**	30,12284	33,93663	34,16646	20,07046	22,14303	22,56658

pp71FL pp71L pp71S

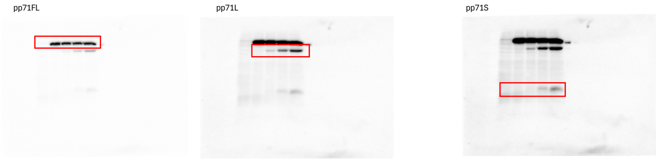

Fig. 4e pp71FL Loading Control

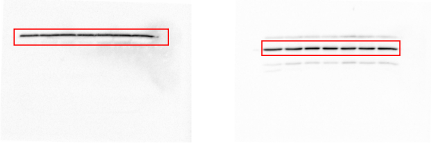

pp71L Loading Control

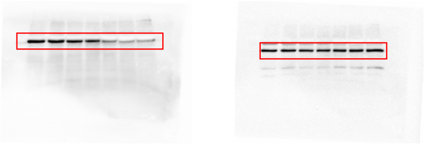

pp71S Loading Control

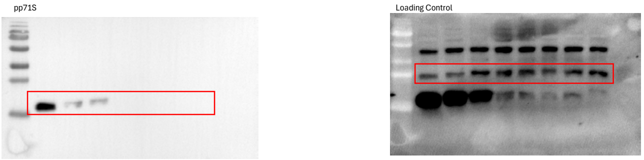

Fig. 4f pp71S Loading Control + anti-ubiquitin

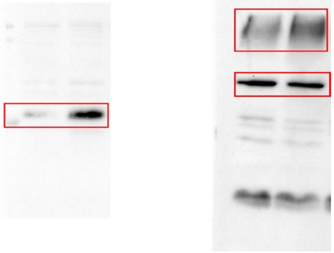

Sup. Fig. 1 In Manuscript

Sup. Fig. 2

HFF - virus			HFF+ virus			NK - virus			NK + virus		
0,8928	1,61926	0,691755	5,681738	10,2983		0,355483	0,271131	0,280169	0,015063	0,01205	
